# Experimental infection of pigs and ferrets with “pre-pandemic,” human-adapted, and swine-adapted variants of the H1N1pdm09 influenza A virus reveals significant differences in viral dynamics and pathological manifestations

**DOI:** 10.1371/journal.ppat.1011838

**Published:** 2023-12-04

**Authors:** Charlotte Kristensen, Helena A. Laybourn, Jeri-Carol Crumpton, Karen Martiny, Ashley Webb, Pia Ryt-Hansen, Ramona Trebbien, Henrik E. Jensen, Jakob N. Nissen, Kerstin Skovgaard, Richard J. Webby, Lars E. Larsen

**Affiliations:** 1 Department of Veterinary and Animal Sciences, University of Copenhagen, Frederiksberg C, Denmark; 2 Department of Biotechnology and Biomedicine, Technical University of Denmark, Kgs. Lyngby, Denmark; 3 Department of Infectious Diseases, St. Jude Children’s Research Hospital, Memphis, TN, United States of America; 4 Department of Virus and Microbiological Special Diagnostics, Statens Serum Institut, Copenhagen S, Denmark; Icahn School of Medicine at Mount Sinai, UNITED STATES

## Abstract

Influenza A viruses are RNA viruses that cause epidemics in humans and are enzootic in the pig population globally. In 2009, pig-to-human transmission of a reassortant H1N1 virus (H1N1pdm09) caused the first influenza pandemic of the 21^st^ century. This study investigated the infection dynamics, pathogenesis, and lesions in pigs and ferrets inoculated with natural isolates of swine-adapted, human-adapted, and “pre-pandemic” H1N1pdm09 viruses. Additionally, the direct-contact and aerosol transmission properties of the three H1N1pdm09 isolates were assessed in ferrets. In pigs, inoculated ferrets, and ferrets infected by direct contact with inoculated ferrets, the pre-pandemic H1N1pdm09 virus induced an intermediary viral load, caused the most severe lesions, and had the highest clinical impact. The swine-adapted H1N1pdm09 virus induced the highest viral load, caused intermediary lesions, and had the least clinical impact in pigs. The human-adapted H1N1pdm09 virus induced the highest viral load, caused the mildest lesions, and had the least clinical impact in ferrets infected by direct contact. The discrepancy between viral load and clinical impact presumably reflects the importance of viral host adaptation. Interestingly, the swine-adapted H1N1pdm09 virus was transmitted by aerosols to two-thirds of the ferrets. Further work is needed to assess the risk of human-to-human aerosol transmission of swine-adapted H1N1pdm09 viruses.

## Author summary

Influenza A viruses (IAVs) have a high evolutionary rate and evolve through genetic drift and reassortment events, elevating their pandemic threat. The last pandemic caused by IAV was a consequence of a spillover of IAV from pigs to humans in 2009 (referred to as H1N1pdm09). The viral and host markers important for host adaptation of IAVs are poorly defined, which makes it difficult to evaluate the pandemic potential of novel swine IAV strains. A method of predicting the risk of human-to-human transmission of IAVs is by using the ferret as an animal model. In this study, we assessed the degree of host adaptation to pigs and ferrets of three different H1N1pdm09 viruses by virological, clinical, and pathological measures. We found that a swine-adapted H1N1pdm09 had the highest viral fitness but the lowest virulence in pigs and that a human-adapted H1N1pdm09 had the highest viral fitness but the lowest virulence in ferrets. In both animal models, we found that an H1N1pdm09 virus resembling a pre-pandemic variant had an intermediary viral fitness but the highest virulence. Our findings indicate that host adaptation is important for the viral fitness and virulence and that an increased viral fitness is not always related with a higher virulence.

## Introduction

Influenza A viruses (IAVs) cause respiratory disease in humans and other animals. IAVs are negative single-stranded RNA viruses with a genome consisting of eight segments. The genome encodes at least 10 essential proteins [1,2]. IAVs are classified into subtypes according to the two surface glycoproteins: hemagglutinin (HA) and neuraminidase (NA) [3]. Three subtypes of IAV (H1N1, H1N2, and H3N2) are enzootic in pigs worldwide [4,5], with H1N1 and H3N2 viruses causing annual epidemics in humans [6]. Sustained genetic differences within IAV subtypes are termed clades [7].

In April 2009, a novel lineage of H1N1 IAV caused the first influenza pandemic of the 21^st^ century. The virus had an HA belonging to the swine influenza virus clade 1A.3.3.2 and was designated H1N1pdm09 [8]. H1N1pdm09 originated from a combination of three IAV lineages circulating in swine: classical swine influenza virus (Csw), Eurasian avian-like swine H1N1 lineage (Clade 1C or EAsw), and the swine triple-reassortant H3N2 lineage (TRsw) [9–12]. The results of retrospective studies strongly indicate that the virus emerged in Mexican swine after multiple reassortment events among the Csw, EAsw, and TRsw lineages [13].

Approximately one month after the first human case, the H1N1pdm09 virus was detected in a pig farm in Canada, a consequence of reverse zoonotic transmission [14] and additional spillover events from humans to swine followed [15]. Subsequently, the H1N1pdm09 viruses became established in swine populations globally [4] resulting in the circulation of “swine-adapted” viruses that differ antigenically from the viruses circulating in humans (human-adapted) [4,16–18].

Swine-adapted H1N1pdm09 viruses have also contributed to a range of reassortment events involving human seasonal IAVs and other enzootic swine IAVs [16, 19–24], thereby increasing viral diversity and the perceived zoonotic and pandemic risk. Knowledge is lacking concerning the basic mechanisms that contribute to the zoonotic potential of swine IAVs, hindering accurate pandemic risk assessments. The zoonotic potential of swine IAV is most likely determined by a combination of viral genetic traits and host-specific factors. There is an urgent need to elucidate the tangled relationship between IAVs in swine and humans and to characterize viruses adapted to each host.

This study compared the pathogenesis, infection dynamics, and impact of swine-adapted, human-adapted, and “pre-pandemic” H1N1pdm09 viruses in experimentally infected pigs and ferrets.

## Materials and methods

### Ethics statement

The pig experiment was performed under biosafety level 2 conditions in accordance with an animal study protocol approved by the Danish Animal Experimentation Council (protocol no. 2020-15-0201-00502). The ferret experiment was performed at St. Jude Children’s Research Hospital (Memphis, TN) in biosafety level 2 enhanced facilities and with the approval of the hospital Animal Care and Use Committee under protocol 428.

### Pig experiment

#### Preparation of virus inoculum

The viruses selected for inoculation were A/swine/Denmark/2017_10298/4_4p1/2017 (H1N1) (swH1N1pdm09), an H1N1pdm09 circulating in pigs (swine-adapted); A/Denmark/238/2020 (H1N1) (huH1N1pdm09), an H1N1pdm09 circulating in humans (human-adapted); and A/Swine/Mexico/AVX-39/2012 (H1N1) (mxH1N1pdm09), a “pre-pandemic” H1N1pdm09 virus (13). The two Danish virus isolates, swH1N1pdm09 and huH1N1pdm09, were propagated and passaged three times in Madin–Darby canine kidney (MDCK) cells. The viruses were stored in aliquots at −80°C until used. The mxH1N1pdm09 virus was kindly provided by Nacho Mena and Adolfo Garcia-Sastre of Mount Sinai School of Medicine and was also passaged in MDCK cells. The titers of the three viruses were determined by tissue culture infectious dose 50% (TCID50) assay in MDCK cells. All virus isolates were diluted in Eagle’s Minimum Essential Medium (MEM) (Gibco) to obtain a TCID50/mL of 10^7^ before inoculation.

#### Study design

**Fig 1:**
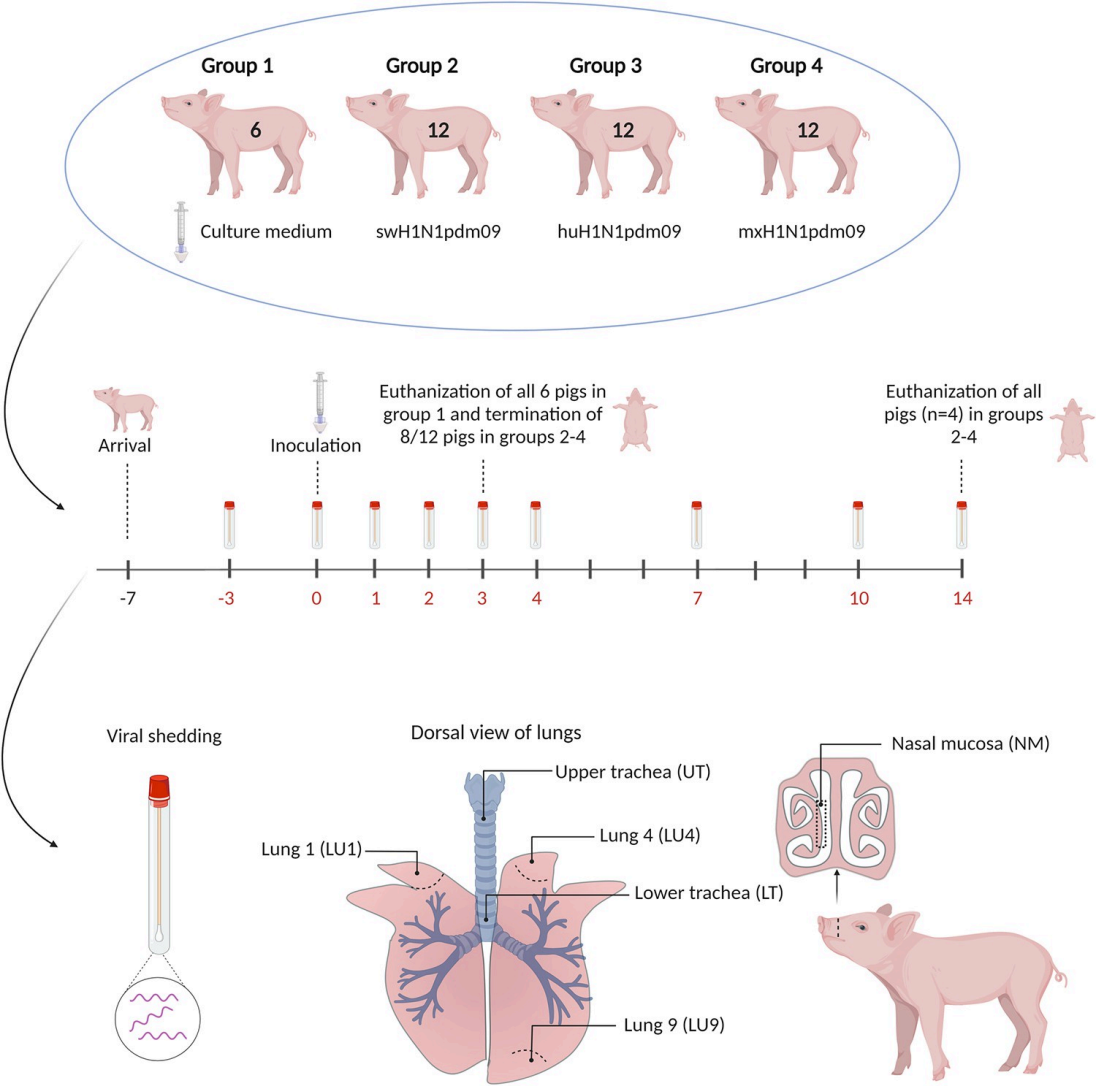
Study design for the pig experiment. DPI = days post inoculation. The tissue specimens included in this paper are illustrated. Created with Biorender.com.

Forty-two 7-week-old Danish Landrace Crossbred pigs (body weight; 7400–12,300 g) that tested negative for swine IAV and antibodies to the virus were included. All pigs were fed with non-pelleted feed (NAG Svinefoder 5) during the study and had *ad libitum* access to water. The pigs were allocated to four groups by minimization (ARRIVE guidelines), with sex and size as nuisance variables, to ensure a balance between the groups (Fig 1). Each group (experimental unit) was housed in a separate isolation unit and had an acclimatization period of 1 week. Groups 2–4 included 12 pigs, whereas group 1 included six pigs. The pigs were enumerated as 1–6, 7–18, 19–30, and 31–42 in groups 1, 2, 3, and 4, respectively. Before inoculation, all pigs were sedated by intramuscular injection (Text S1). All pigs in groups 2–4 were inoculated intranasally (in the right nostril) with 3 mL of virus, with a titer of 10^7^ TCID50/mL, by using MAD Nasal intranasal mucosal atomization devices (Teleflex, Morrisville, NC, cat. no. MAD100). Group 1 pigs (the control group) were mock infected intranasally with cell culture medium only, again using the mucosal atomization devices. Pigs in groups 2, 3, and 4 were inoculated with swH1N1pdm09, huH1N1pdm09, and mxH1N1pdm09, respectively. Control pigs and eight pigs from each IAV-infected group were euthanized 3 days post inoculation (DPI), whereas the remaining pigs (pigs 15–18 from group 2, 27–30 from group 3, and 39–42 from group 4) were euthanized on day 14 post inoculation.

#### Observations and sampling

The pigs were weighed at 0, 3, and 14 DPI. Body temperature measurements, nasal swabs, and blood samples were collected at −3, 0, 1, 2, 3, 4, 7, 10, and 14 DPI. At 0, 3, and 14 DPI, the samples were collected after sedation but before inoculation and euthanasia, except for those collected from the four pigs in each IAV-infected group at 3 DPI, which lived until 14 DPI. More details of the observation and sampling can be found in Text S1.

On the day of inoculation (0 DPI), all pigs were tested by nasal swabbing for porcine circovirus type 2 (PCV2), porcine reproductive and respiratory syndrome virus (PRRSV) types 1 and 2, and *Mycoplasma hyopneumoniae* as described previously [25].

#### Necropsy and histopathology

Pigs were sedated before euthanasia, following the same protocol described in Text S1. They were exsanguinated by cutting the axillary artery. At necropsy, gross lesions were recorded, lung lesions compatible with IAV infection (red or dark red lobular atelectasis) were sketched, and the lungs were photographed. Lesion severity was graded by the amount of atelectasis in each lung lobe: 0: no atelectasis, 1: > 0–30%, 2: ≥ 30–60%, 3: ≥ 60% as described previously [26], and the highest atelectasis score for all lung lobes was chosen to provide one gross lesion score for each pig. Furthermore, the number of affected lung lobes was registered. Specimens of tissues mentioned in S1 Table were collected from each pig for IAV quantification by RT-qPCR, sequencing, and histopathological examination. All samples were collected aseptically. Tissue specimens for IAV quantification and sequencing were placed in 1.5-mL Eppendorf tubes and stored at −80°C until analyzed. Specimens for histopathology were fixed in 10% neutral-buffered formalin for a week, embedded in paraffin wax, sliced into 2–3 μm sections, and stained with hematoxylin and eosin (H&E). Histologically, all lung tissues (LU1, LU4, and LU9) from animals at 3 DPI were graded using a modified scoring scheme [27], briefly, the amount of peribronchial/peribronchiolar infiltration, bronchiolar luminal exudate and alveolar infiltration was evaluated (S2 Table). The highest histopathological score for all lung tissues was chosen to provide one histopathological score for each pig. Based on the quality of the tissue preparation, histological evaluations were performed of the nasal mucosa and upper trachea of one pig from the control group at 3 DPI, of two pigs from each inoculated group at 3 DPI, and of two pigs from each inoculated group at 14 DPI. Immunohistochemical staining targeting cytokeratin (for epithelial cells) was performed on selected representative sections to confirm hyperplasia of type II pneumocytes [28].

### Ferret study

#### Preparation of virus inoculum

The viruses used for inoculating ferrets were identical to those used in the swine experiment and were propagated as described above. All virus isolates were diluted in sterile PBS before inoculation. A back titration showed that the dose unintentionally varied between the groups so the swH1N1pdm09 strain had a titer of 10^5^ TCID50/mL, the huH1N1pdm09 strain 5 × 10^4^ TCID50/mL, and the mxH1N1pdm09 strain 5 × 10^5^ TCID50/mL.

#### Study design

In total, the study used 36 male ferrets (aged 9–23 weeks; body weight: 0.97–1.48 kg) (Triple F Farms) that were negative for IAV antibodies by the HI test. Donor and recipient ferrets were housed separately. After 1 week of acclimatization, three donor ferrets from each group were sedated with 4% isoflurane and inoculated intranasally with 0.5 mL of virus (250 μL of virus diluted in sterile PBS in each nostril), using a syringe. Ferrets from group 1 were inoculated with swH1N1pdm90, those from group 2 with huH1N1pdm09, and those from group 3 with mxH1N1pdm09. After inoculation, donor ferrets were housed in separate cages. Data from donor ferrets were not included in this study. Three additional ferrets (henceforth referred to as “inoculated ferrets”) were inoculated with each virus and euthanized at 3 DPI. The group 1 inoculated ferrets were numbered 1–3, the group 2 ferrets were numbered 4–6, and the group 3 ferrets were numbered 7–9.

**Fig 2:**
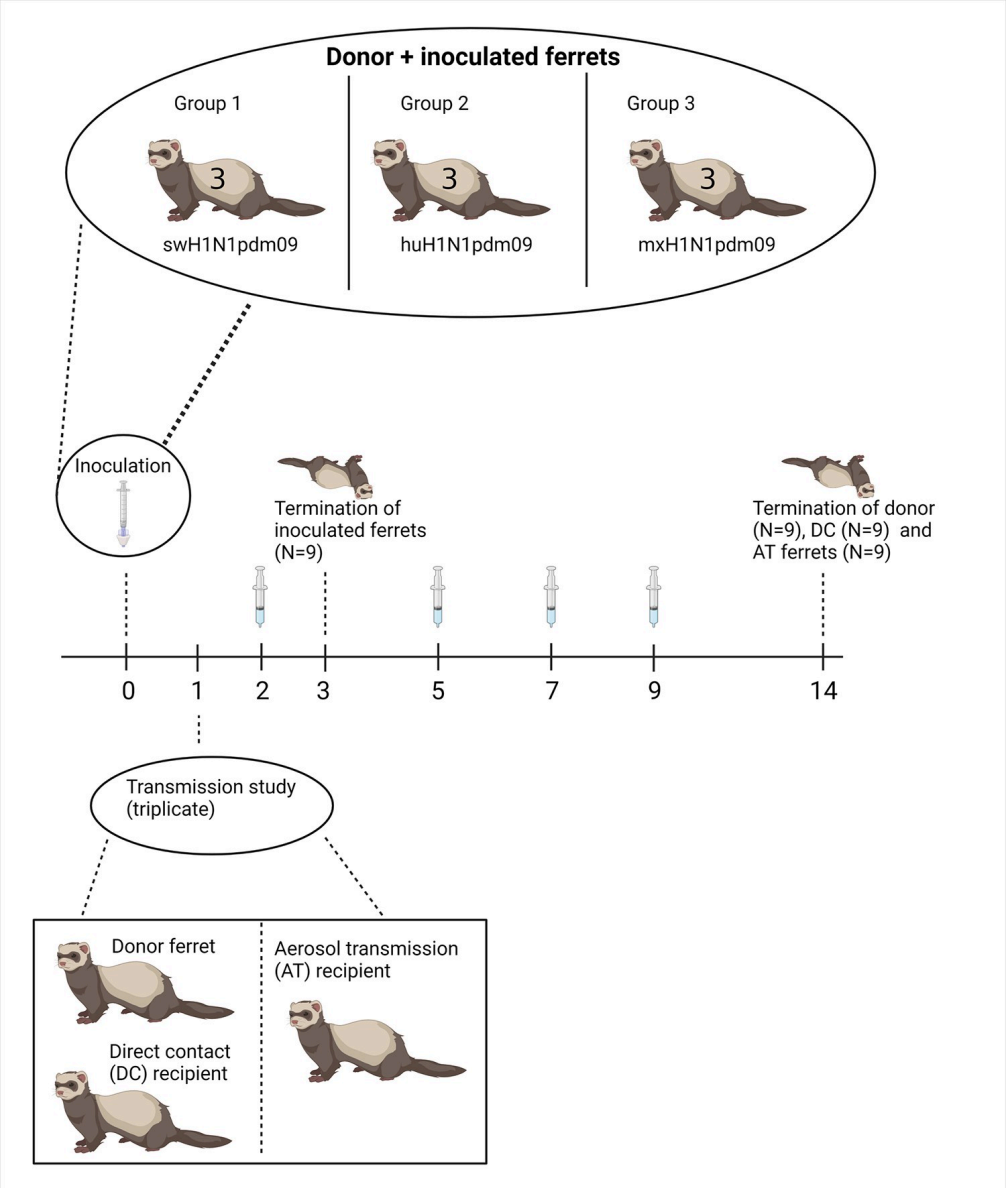
Study design for the ferret experiment. DPI = days post inoculation. Created with Biorender.com.

Twenty-four hours after inoculation, each donor ferret was co-housed with one naïve ferret (DC recipient). DC ferrets were assigned the numbers 10–12 in group 1, 13–15 in group 2, and 16–18 in group 3. Additionally, naïve ferrets were placed in a cage adjacent to donor and DC ferrets separated by double-layered perforated dividers to allow respiratory droplet transmission to assess aerosol transmission (AT recipient). AT ferrets were assigned the numbers 19–21 in group 1, 22–24 in group 2, and 25–27 in group 3. The ferret experiment was conducted in triplicates for each strain (one donor, one DC recipient, and one AT recipient). Donor, DC and AT ferrets were euthanized at 14 DPI (Fig 2).

#### Observations and sampling

Body weight and temperature were measured at 0, 2, 5, 7, and 9 DPI. Nasal washes for quantification of IAV by real-time RT-PCR were performed at 2, 5, 7, and 9 DPI after the administration of 0.4 mL of ketamine I.M as described previously [29], and the samples were stored at −80°C until analyzed.

#### Necropsy and histopathology

Ferrets were euthanized by intracardiac administration of 1.0 mL of Euthasol after sedation with 0.4 mL of a ketamine/xylazine mixture. Specimens of organs mentioned in S1 Table were collected from inoculated ferrets at 3 DPI and from DC ferrets at 14 DPI for IAV quantification by RT-qPCR, sequencing, and histopathological examination. Specimens collected for IAV quantification and sequencing were stored at −80°C until analyzed. Specimens for histopathological examination were processed as for the pig tissues. Lung sections from all inoculated and DC ferrets were examined together with one section of the nasal turbinates and trachea from each group of inoculated and DC ferrets. These sections were selected based on the quality of the histopathological preparation.

### Serological tests

ELISAs for anti-IAV antibodies were performed using the IDEXX Influenza A ELISA Kit (IDEXX laboratories, Westbrook, ME) as described previously [30]. ELISA results were calculated as a signal-to-noise ratio (S/N), using the cut-off recommended by the vendor.

### RNA extraction and quantification of IAV by real-time RT-PCR

Nasal swab samples were vortex mixed for 10 s and centrifuged for 3 min at 9651 ×* g*. For each sample, 200 μL of the supernatant was mixed with 400 μL of RLT buffer (QIAGEN, Hilden, Germany) containing 2-mercaptoethanol (ME) (Merck, Darmstadt, Germany). Nasal mucosa, tracheal tissues, and lung tissues (70 mg of each) were lysed by bead beating in a TissueLyser LT (QIAGEN) for 3 min at 30 Hz in 1400 μL of RLT buffer and centrifuged for 3 min at 9651 × *g*. After centrifugation, 600 μL of the supernatant was used for RNA extraction, which was performed as described previously [31]. Extracted RNA was eluted in 60 μL of RNase-free water and stored at −80°C.

The PCR assay used to detect IAV was an in-house modified version of a real-time RT-PCR assay targeting the matrix gene [32]. The primers RimF (5′-CTT CTA ACC GAG GTC GAA ACG-3′) and RimR (5′-AGG GCA TTT TGG ACA AAK CGT CTA-3′) and the dual-labeled probe MAprobeDL (5′-FAM CCC AGT GAG CGA GGA CTG CAG CGT BHQ-1-3′) were used. The master mix had a final volume of 25 μL, containing 3 μL of RNA solution, 13 μL of SensiFAST Probe No-ROX One-Step Mix (Meridian Bioscience, Boxtel, The Netherlands), 0.55 μL of RiboSafe RNase Inhibitor (Meridian Bioscience), 0.28 μL of reverse transcriptase (Meridian Bioscience), 7 μL of RNase-free water, and 2 μL of primer–probe mix (0.08 μL of each primer [0.4 μmol of each], 0.06 μL of probe [0.3 μmol], and 1.78 μL of RNase-free water). The RT-qPCR was performed on a Rotor-Gene Q platform (QIAGEN, Hilden, Germany) using the following program: 30 min at 50°C then 2 min at 95°C, followed by 40 cycles of 15 s at 95°C, 15 s at 55°C, and 20 s at 72°C. Positive and negative controls were included in all runs. Nucleic acid from IAVs was quantified in nasal swabs/washes, nasal mucosa, tracheal tissue, and lung tissue based on a 10-fold dilution series of the target sequence with known copy numbers.

### Viral titration

In pigs, the nasal swab specimen with the highest copies/ml collected 2 or 3 DPI and the lung specimen with the highest copies/ml collected at 3 DPI were homogenized, sterile filtered, and titrated in MDCK cells. In ferrets all nasal washes collected and tissues collected from inoculated ferrets at 3 DPI were homogenized and titrated in MDCK cells. The TCID50/ml was calculated by using the Reed–Muench method [33].

### Sequencing of virus and phylogeny

Full-genome sequencing was performed on the inoculum virus. RNA was extracted as described previously, and all segments were amplified in one tube by using a previously published protocol [31]. Next-generation sequencing and generation of consensus sequences and MUSCLE alignment was performed as described previously [31]. No genetics changes in the inoculum strains were observed after the passages in MDCK cells.

A maximum-likelihood tree of HA gene segments was generated with selected reference sequences and by using ModelFinder [34] and IqTree [35]. The best-fit model was the substitution model: K3Pu+F+I. Reference sequences originating from the human H1N1pdm09 subtype from 2009–2020 were obtained from annual reports of the European Centre for Disease Prevention and Control (ECDC). A pairwise comparison of the coding nucleotide HA sequences of the inoculum strains was performed using CLC Main Workbench version 22.0 (QIAGEN, Aarhus, Denmark) to measure the percentage identity.

### Statistical analysis

A blinded inter-observer agreement study of the histopathological scoring was performed. Descriptive statistics for nasal shedding (measured with nasal swabs or washes) by pigs at 1–12 DPI (n  =  4), inoculated ferrets (n  =  3), DC ferrets (n  =  3), and AT ferrets (n  =  3) consisted of the median and the interquartile range (25%–75%). The total amount of virus shed over time (the total viral load) was calculated as the area under the curve (AUC) for nasal swab samples collected from pigs at 1–3 DPI (n  =  12), nasal washes from DC ferrets at 2–9 DPI (n  =  3), and nasal washes from AT ferrets at 2–9 DPI (n  =  3).

No animals were excluded from the experiments, but the nasal wash for ferret no. 10 in the AT swH1N1pdm09 group at 9 DPI was missing. To test for a normal distribution of the data, a Shapiro–Wilk test or an Anderson–Darling test and an analysis of QQ plots in RStudio were performed. In pigs, the differences in body weight gain were examined by two-way ANOVA with a Tukey correction of the *P* values, and the results are reported as the mean ± SD with a 95% confidence interval (CI) for the differences. There was a lack of normal distributions for viral shedding at 1–3 DPI (n  =  12), the total viral load (AUC) at 1–3 DPI (n  =  12), the severity of lung lobe lesions at 3 DPI (n  =  8), and the number of affected lung lobes at 3 DPI (n  =  8). These data points are, therefore, reported as medians with the interquartile ranges (25%–75%), and they were analyzed by Kruskal–Wallis tests and by a post hoc Dunn test with a pairwise comparison and Bonferroni correction of the *P* values with a 95% CI for the differences in mean ranks between the groups. Statistics of the viral shedding and viral load were performed on positive samples only. Correlation was investigated by the calculation of Spearman’s correlation coefficient. Statistical analyses were performed in RStudio Team (http://www.rstudio.com) or in GraphPad Prism version 9.0.0 for Windows (GraphPad Software, San Diego, California USA, www.graphpad.com) with *P* values of 0.05 or less being considered to indicate statistical significance. Data were illustrated using GraphPad Prism.

## Results

### Genetic characterization

A maximum-likelihood tree of the H1pdm09 study viruses and selected reference viruses is presented in S1 Fig. Although the mxH1N1pdm09 virus was isolated in 2012, 3 years after being first detected in humans, phylogenetic analyses strongly support this strain being a “pre-pandemic” version of the virus [13]. The tree also illustrates the genetic differences between the huH1N1pdm09 and swH1N1pdm09 viruses. The swH1N1pdm09 virus clusters within the swine H1N1pdm09 clade (1.A.3.3.2) [16], whereas the huH1N1pdm09 virus clusters with the human clade 6B, alongside a few swine H1N1pdm09 isolates. The huH1N1pdm09 inoculum strain had 93.6% and 93.1% nucleotide identity of the HA gene when compared with the swH1N1pdm09 and mxH1N1pdm09 inoculum strains, respectively. The swH1N1pdm09 virus had 92.1% nucleotide identity of the HA gene when compared with the mxH1N1pdm09 strain.

### Pig study

#### Clinical signs were more severe in pigs in the mxH1N1pdm09 group

Gains in body weight and rectal temperature measurements are summarized in Table 1, with the clinical signs being detailed in S1 Text. All pigs tested negative for PCV2, PRRSV types 1 and 2, and *Mycoplasma hyopneumoniae* by nasal swabbing at 0 DPI. Pigs in the mxH1N1pdm09 and huH1N1pdm09 groups had gained significantly less weight at 3 DPI when compared to control pigs (*P* ≤ 0.001 and *P* ≤ 0.01, respectively). Furthermore, weight gain was significantly higher in the swH1N1pdm09 group than in the mxH1N1pdm09 group (*P* ≤ 0.05). Sneezing was observed in two pigs in the swH1N1pdm09 group and lethargy in three pigs in each of the IAV-inoculated groups. A weak negative correlation between maximal increase in body temperatures from baseline and weight gain from 0 to 3 DPI was observed (r = -0.47, (95% CI: -0.68; -0.18), *P* < 0.01).

**Table 1:** Summary of clinical observations from the pig experiment*.

Group	Rectal temperature^1^	Mean weight gain: 0 to 3 DPI (kg)^2^	Mean weight gain: 0 to 14 DPI (kg)^3^
**Control**	0.5	1.38 ± 0.28^a^	-
**swH1N1pdm09**	1.3	1.02 ± 0.41^ac^	4.27 ± 0.67
**huH1N1pdm09**	1.6	0.71 ± 0.24^bc^	4.13 ± 0.87
**mxH1N1pdm09**	1.8	0.52 ± 0.43^b^	3.58 ± 1.69

*Means followed by a different superscript letter are significantly different by two-way ANOVA test (*P* < 0.05).

^1^ Median maximal increase of baseline body temperatures

^2^ Mean weight gain of pigs from day 0 to 3 DPI ± SD, N  =  42

^3^ Mean weight gain of pigs from day 0 to 14 DPI ± SD, N  =  12

#### Pigs in the swH1N1pdm09 group had significantly higher viral loads

**Fig 3:**
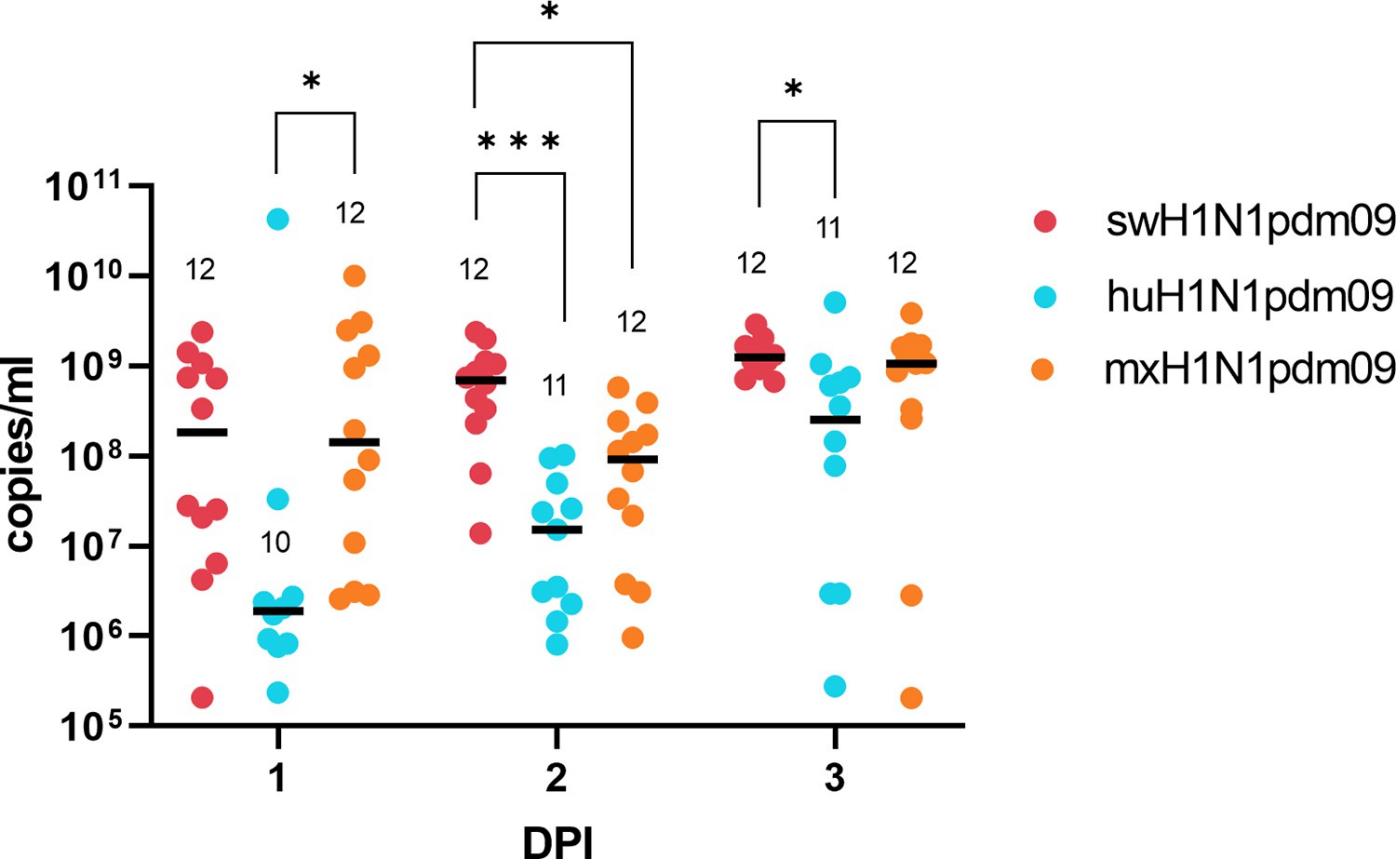
Scatterplot of viral shedding as detected by nasal swabbing in pigs on days post inoculation (DPI) 1 to 3. Each pig is represented by a dot colored according to their group. Black lines represent the median viral shedding of positive samples. The number of positive samples out of the total number of pigs in the group (n  =  12) is presented above each cluster of dots. (*/***) denotes a significant difference in viral shedding between groups.

No shedding of IAV was detected at 0 DPI in any study animal. The viral load was significantly higher in the mxH1N1pdm09 group than in the huH1N1pdm09 group at 1 DPI (*P* < 0.05) (Fig 3). The viral load was also significantly higher in the swH1N1pdm09 group than in the huH1N1pdm09 group at 2 and 3 DPI (*P* < 0.001 and *P* < 0.05, respectively). Furthermore, the viral load was significantly higher in the swH1N1pdm09 group than in the mxH1N1pdm09 group at 2 DPI (*P* < 0.05). The total viral load expressed as the median area under the curve (AUC) differed among the groups, being 1.95 × 10^9^ (1.46–2.51 × 10^9^) copies/mL for the swH1N1pdm09 group, 2.33 × 10^8^ (1.98 × 10^7^–4.44 × 10^8^) copies/mL for the huH1N1pdm09 group, and 1.19 × 10^9^ (2.47 × 10^8^–2.32 × 10^9^) copies/mL for the mxH1N1pdm09 group (S2A Fig). The total viral load was significantly higher in the swH1N1pdm09 group than in the huH1N1pdm09 group (*P* < 0.05).

In all groups, the median viral shedding as detected by nasal swabbing was highest at 4 DPI: 2.10 × 10^9^ (3.59  × 10^8^–3.93 × 10^9^) copies/mL for the swH1N1pdm09 group, 6.25 × 10^8^ (2.67 × 10^8^–1.08 × 10^9^) copies/mL for the huH1N1pdm09 group, and 1.61 × 10^9^ (9.76  × 10^8^–2.26 × 10^9^) copies/mL for the mxH1N1pdm09 group (S2B Fig). Infectious virus was isolated in nasal swabs from 12/12 pigs in the swH1N1pdm09 group, 9/12 pigs in the mxH1N1pdm09 group, and 8/12 pigs in the huH1N1pdm09 group.

#### Most pigs seroconverted

The NP ELISA for IAV at 14 DPI showed that all four pigs in the mxH1N1pdm09 group, three of four pigs in the huH1N1pdm09 group, and two of four pigs in the swH1N1pdm09 group had seroconverted.

#### The highest viral loads were in lungs of pigs inoculated with mxH1N1pdm09 or swH1N1pdm09 virus

**Fig 4:**
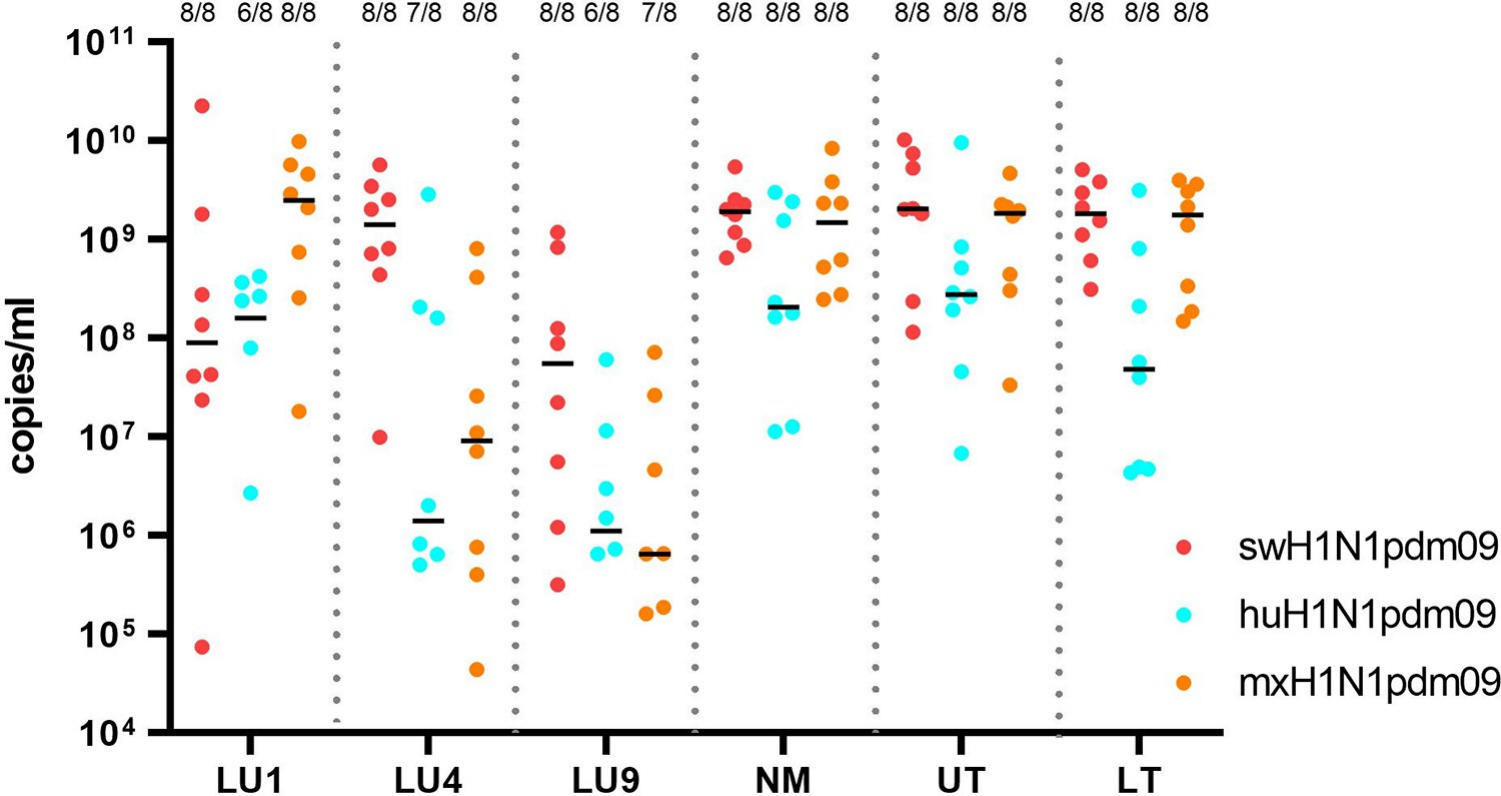
Viral load in porcine lung tissues (LU1, LU4, LU9), nasal mucosa (NM), upper trachea (UT), and lower trachea (LT) collected at 3 DPI. Black lines represent the median viral load in the different tissue specimens. The number of positive samples out of the total number of pigs in the groups (n  =  8) is presented above each cluster of dots as a fraction.

Pigs in the mxH1N1pdm09 group had the highest viral load in the lungs (LU1) [1.44 × 10^9^ 6.03 × 10^8^–3.35 × 10^9^) copies/mL], whereas pigs in the swH1N1pdm09 and huH1N1pdm09 groups had the highest viral loads in the upper trachea [2.03 × 10^9^ (1.42 × 10^8^–5.77 × 10^9^) copies/mL and 2.76 × 10^8^ (1.56 × 10^7^–2.16 × 10^8^) copies/mL, respectively] (Fig 4). Infectious virus was detected in lung tissues at 3 DPI from 8/8 pigs, 6/8 pigs and 8/8 pigs in the swH1N1pdm09, huH1N1pdm09 and mxH1N1pdm09 groups, respectively. A strong positive correlation was found between viral lung titers at 3 DPI and the maximal increase in body temperatures from baseline in pigs from the mxH1N1pdm09 group (r = 0.81, *P* < 0.02).

A few pigs in each IAV-infected group were PCR positive for viral RNA in their lungs, nasal mucosa, and upper and lower trachea at 14 DPI.

#### Lesions were significantly more severe in mxH1N1pdm09-infected pigs

Pulmonary emphysema was observed in some pigs, including some controls; otherwise, no gross lesions were observed in the controls. Almost all pigs inoculated with IAV showed multifocal, lobular, atelectasis with a varying degree of redness (S3 Fig). However, no gross lesions were observed at 3 DPI in one swH1N1pdm09-inoculated pig or in two pigs inoculated with huH1N1pdm09 virus. Four pigs developed pleuritis and pericarditis, and one pig in the swH1N1pdm09 group also had purulent rhinitis. The severity of atelectasis and the number of affected lung lobes differed among the groups at 3 DPI (Table 2). Atelectasis was significantly more severe in the mxH1N1pdm09 group than in the huH1N1pdm09 group (*P* < 0.05). Furthermore, there were significantly fewer affected lung lobes in the swH1N1pdm09 group than in the mxH1N1pdm09 group (*P* < 0.05). At 14 DPI, one pig in the swH1N1pdm09 group and two pigs in the huH1N1pdm09 group showed mild atelectasis, whereas none of the pigs in the mxH1N1pdm09 group had lesions.

**Table 2:** * Group median macroscopic scores (+ 25% and 75% percentiles) from inoculated pigs at 3 DPI (n  =  8)[Table-fn t002fn001].

Group	Severity of affected lung lobes^1^	No. of affected lung lobes
**swH1N1pdm09**	2.00 [1.00; 3.00]^ab^	1.00 [1.00; 2.00]^a^
**huH1N1pdm09**	1.50 [0.25; 2.00]^a^	2.00 [0.25; 2.75]^ab^
**mxH1N1pdm09**	3.00 [2.25; 3.00]^b^	3.50 [2.25; 4.00]^b^

*Medians followed by a different superscript letter are significantly different by a Kruskal–Wallis test (*P* ***< ***0.05).

^1^ Median [25%;75% percentiles] of the highest atelectasis score obtained from affected lung lobes from each pig.

#### Histopathological changes were more severe in mxH1N1pdm09-infected and swH1N1pdm09-infected pigs

**Fig 5:**
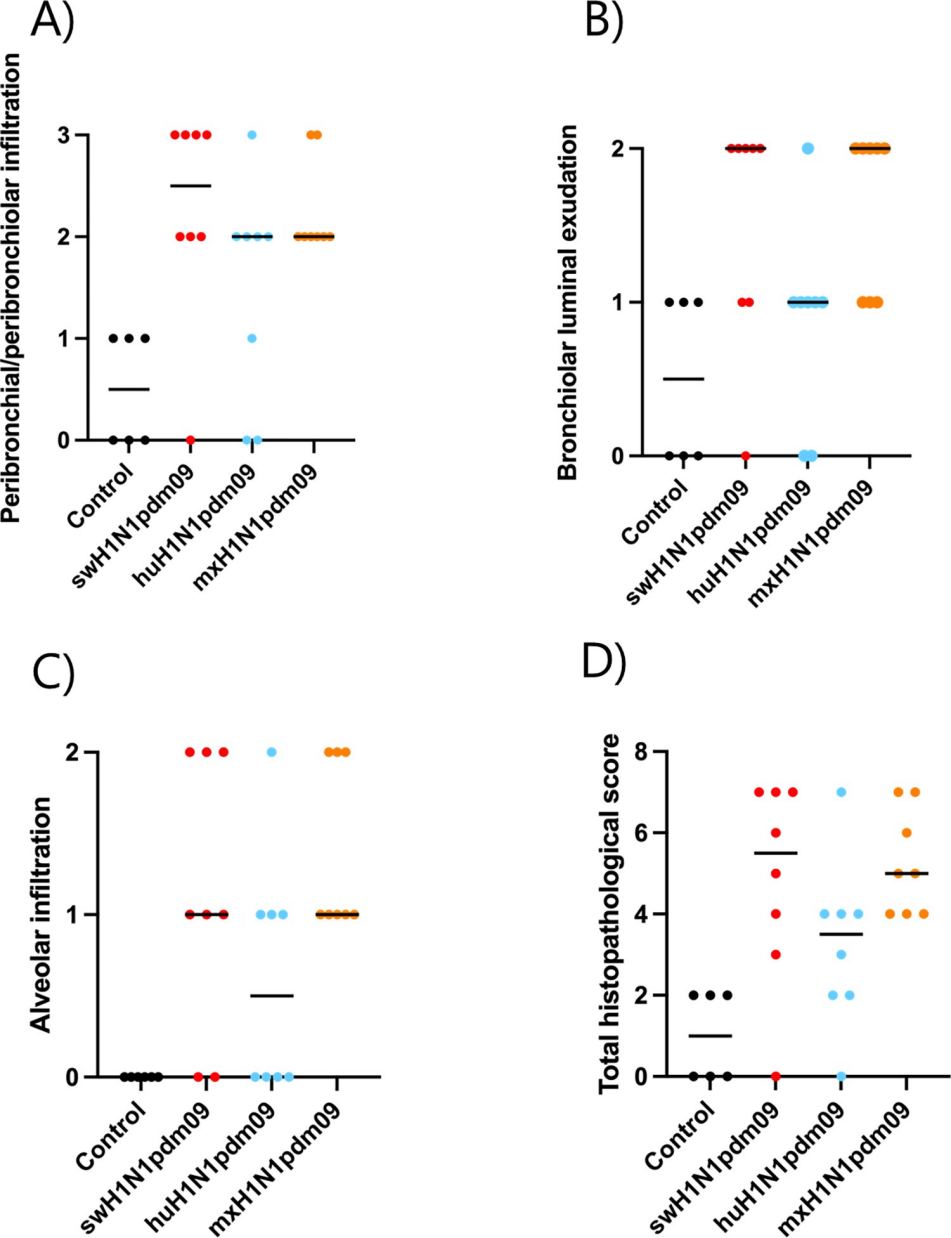
The group median histopathological scores for each category for pigs at 3 DPI. The categories were as follows: peribronchial/peribronchiolar infiltrates (none, few [<10%], many [10%–50%], majority or all [>50%]), bronchial luminal exudate (none, minimal, heavy), and alveolar infiltrates (none, minimal, heavy) (see S2 Table).

**Fig 6:**
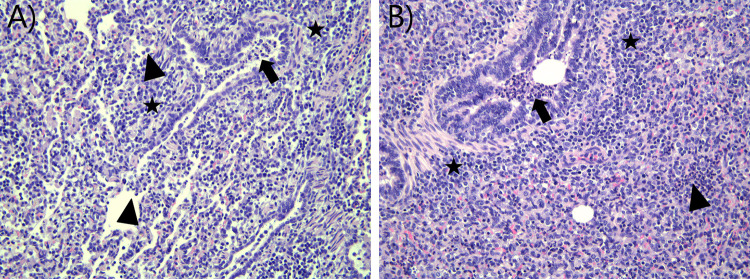
Histopathological changes in infected pigs at 3 days post inoculation. A) Lung tissue from an mxH1N1pdm09-infected pig at 3 DPI with a histopathological score of 5, showing bronchiolitis with exudation dominated by neutrophils and some mononuclear cells (arrow), patchy infiltration in the alveoli (arrowheads), and peribronchiolar infiltration by mononuclear cells (stars). B) Lung tissue from an swH1N1pdm09-infected pig at 3 DPI with a histopathological score of 7, showing bronchiolitis with massive exudation of neutrophils and necrotic debris (arrow), infiltration of neutrophils in the alveoli (arrowhead), and marked peribronchiolar infiltration by mononuclear cells (stars). H&E stained.

Some histopathological background lesions were found in control pigs and were, therefore, not taken into account in the histopathological grading (S1 Text and S4A and S4B Fig). Pulmonary lesions were similar in all infected groups; however, their severity and the percentage of bronchi/bronchioles affected differed between and within groups (Figs 5, 6A and 6B and S3 Table). The total histopathological scoring was evaluated by a blinded inter-observer agreement study with weighted kappa coefficients (κW) of 0.67, which is considered to represent substantial agreement [36]. The control pig showed no inflammation in the nasal mucosa and upper trachea at 3 DPI. All IAV-inoculated pigs showed acute, moderate, suppurative, necrotizing rhinitis (S4C Fig), and all pigs, except one mxH1N1pdm09-infected pig, had acute, necrotizing tracheitis of varied severity at 3 DPI (S4D and S4E Fig).

**Fig 7:**
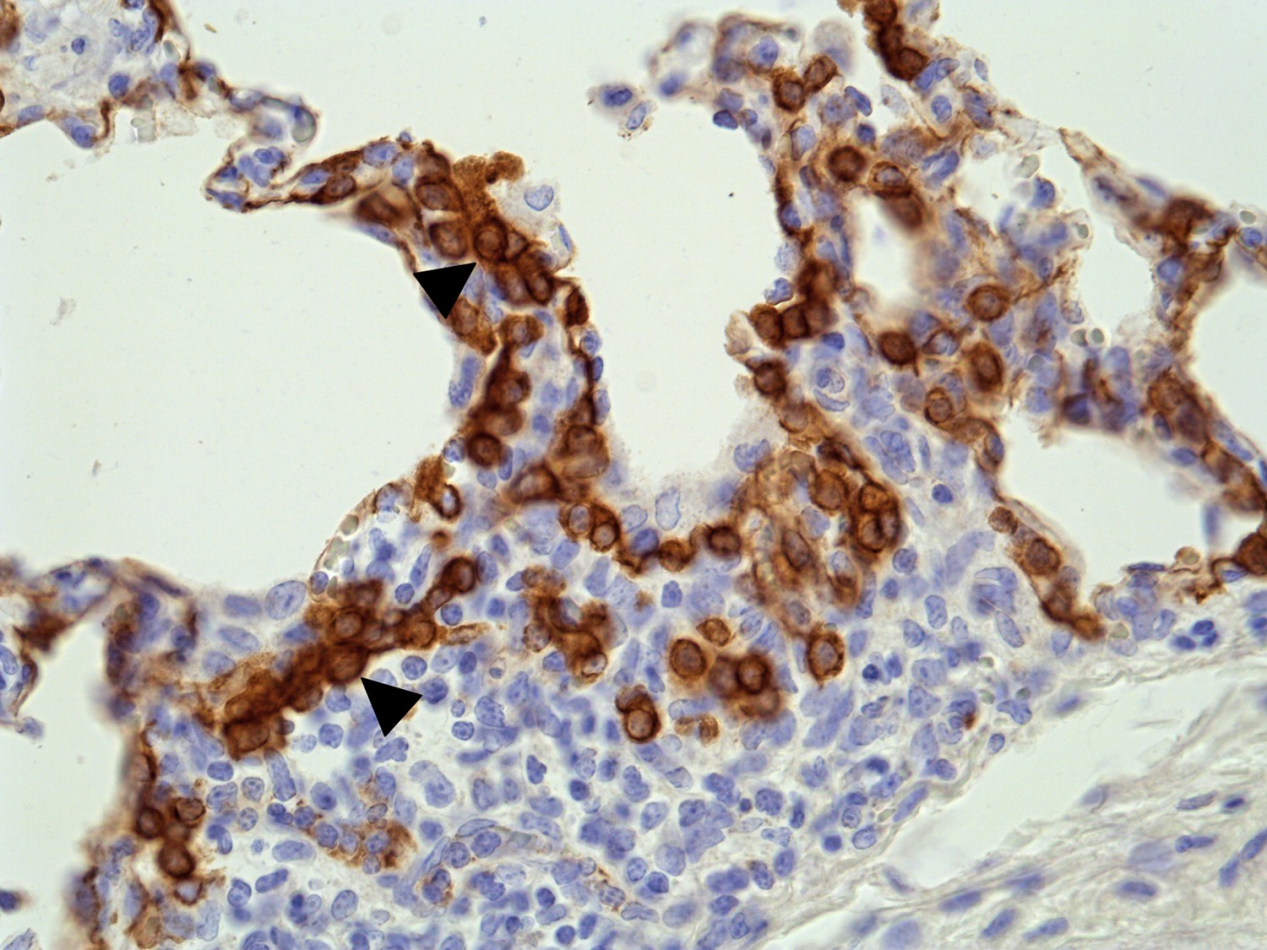
Lung tissue from a swH1N1pdm09-infected pig at 14 DPI. Moderate hyperplasia of cytokeratin positive type II pneumocytes (arrowheads) is demonstrated by immunohistochemical staining for cytokeratin.

Background lung lesions similar to those observed in the control pigs, together with a few additional changes, were found in some infected pigs from each group at 14 DPI (S4F Fig and S3 Table). Moderate hyperplasia of type 2 pneumocytes was observed at 14 DPI in all IAV-infected groups (Fig 7). Only mild or no lesions of the nasal mucosa and tracheal tissues were observed at 14 DPI. A more detailed description of the nasal and tracheal lesions at 3 and 14 DPI is presented in S3 Table.

### Ferret study

#### Clinical impact varied among inoculated, DC, and AT ferrets

No ferrets developed clinical signs or fever (defined as a temperature above 40°C), but the inoculated and DC ferrets had increased body temperatures at 2 DPI (S5A and S5B Fig and S1 Text). In inoculated ferrets, the median body temperature increase was similar across groups, but the highest body temperature increase was observed in the huH1N1pdm09 group and the lowest in the mxH1N1pdm09 group (S5A Fig and S4 Table). In DC ferrets, the highest increase in body temperatures at 2 DPI was in the swH1N1pdm09 and mxH1N1pdm09 groups, as compared with the huH1N1pdm09 group (S5B Fig). The body temperatures of DC ferrets had decreased almost to or below baseline at 5, 7, and 9 DPI, whereas the body temperatures of the AT ferrets decreased or remained stable during the study (S5C Fig).

The average weight loss in inoculated and DC ferrets was higher in the mxH1N1pdm09 group than in the huH1N1pdm09 and swH1N1pdm09 groups (S4 Table and S6A and S6B Fig). In contrast, the weight loss in AT ferrets was highest in the huH1N1pdm09 group and lowest in the swH1N1pdm09 and mxH1N1pdm09 groups (S6C Fig).

#### The huH1N1pdm09-infected ferrets had the highest viral load

**Fig 8:**
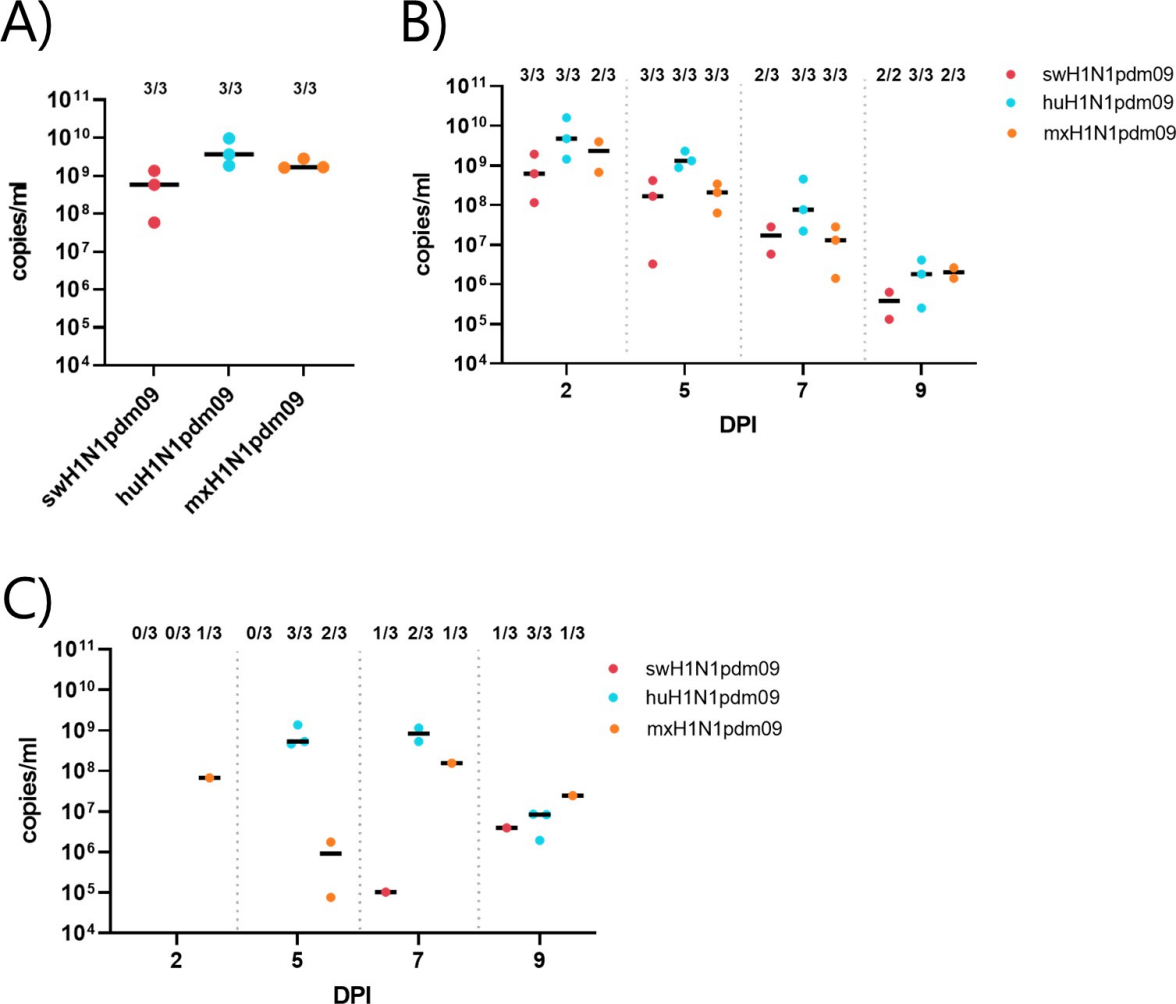
Scatterplot of viral shedding as detected by nasal washes in ferrets. A) Scatterplot of viral shedding as detected in nasal washes collected from inoculated ferrets at 2 days post inoculation (DPI). B) Scatterplot of viral shedding as detected in nasal washes collected from DC ferrets at 2, 5, 7, and 9 DPI. One 9-DPI sample from the swH1N1pdm09 group was missing. C) Scatterplot of viral shedding as detected in nasal washes collected from AT ferrets at 2, 5, 7, and 9 DPI. Black lines represent the median viral shedding for positive samples. The number of positive samples out of the total number of samples in the group is presented above each cluster of dots as a fraction.

The viral load was measured in all inoculated ferrets at 2 DPI. In the inoculated ferrets, the viral shedding was higher in the huH1N1pdm09 group than in those in the swH1N1pdm09 and mxH1N1pdm09 groups (Fig 8A and S4 Table). Infectious virus was detected in nasal washes from all inoculated-ferrets at 2 DPI.

By using RT-qPCR, all ferrets in the huH1N1pdm09 DC group tested positive for IAV on all sampling days, whereas all swH1N1pdm09 DC ferrets were positive at 2, 5, and 9 DPI and all mxH1N1pdm09 DC ferrets were positive at 5 and 7 DPI (Fig 8B). On all sampling days, the highest viral load was detected in the huH1N1pdm09 DC group, peaking at 4.73 × 10^9^ (3.08 × 10^9^–1.04 × 10^10^) copies/mL at 2 DPI. The viral load in the swH1N1pdm09 and mxH1N1pdm09 DC ferrets also peaked at 2 DPI, reaching 6.14 × 10^8^ (3.64 ×10^8^–1.27×10^9^) copies/mL in the swH1N1pdm09 ferrets and 2.31 × 10^9^ (1.49 × 10^9^–3.13 × 10^9^) copies/mL in the mxH1N1pdm09 ferrets. Infectious virus was detected in nasal washes from all DC ferrets at 2 and 5 DPI. In general, DC ferrets exhibited higher viral shedding at 2 DPI when compared with inoculated ferrets.

By using RT-qPCR, all huH1N1pdm09 AT ferrets tested positive for IAV at 5 and 9 DPI, +- one ferret in the huH1N1pdm09 group tested negative for IAV at 7 DPI. Two ferrets in the swH1N1pdm09 group tested positive at different time points (7 DPI and 9 DPI), whereas one ferret remained negative for IAV throughout the study period. One mxH1N1pdm09 AT ferret tested positive for IAV at 2 and 5 DPI, and another tested positive for IAV at 5, 7, and 9 DPI; the last mxH1N1pdm09 AT ferret remained negative throughout the study (Fig 8C). Infectious virus was detected in nasal washes from all AT huH1N1pdm09 ferrets at 2 and 5 DPI and in one ferret at 7 DPI, whereas this was only observed in one nasal wash from an AT mxH1N1pdm09 ferret and in none of the AT swH1N1pdm09 ferrets. The numbers of DC and AT ferrets that tested positive at least once during the study measured by both RT-qPCR and TCID50 are summarized in S4 Table.

The total viral load of DC and AT ferrets as determined from nasal washes and RT-qPCR is expressed as the median AUC (S6D and S6E Fig). In general, the total viral load was highest in the huH1N1pdm09 groups, followed by the mxH1N1pdm09 groups and then the swH1N1pdm09 groups.

#### The highest viral lung load was found in mxH1N1pdm09 inoculated ferrets

**Fig 9:**
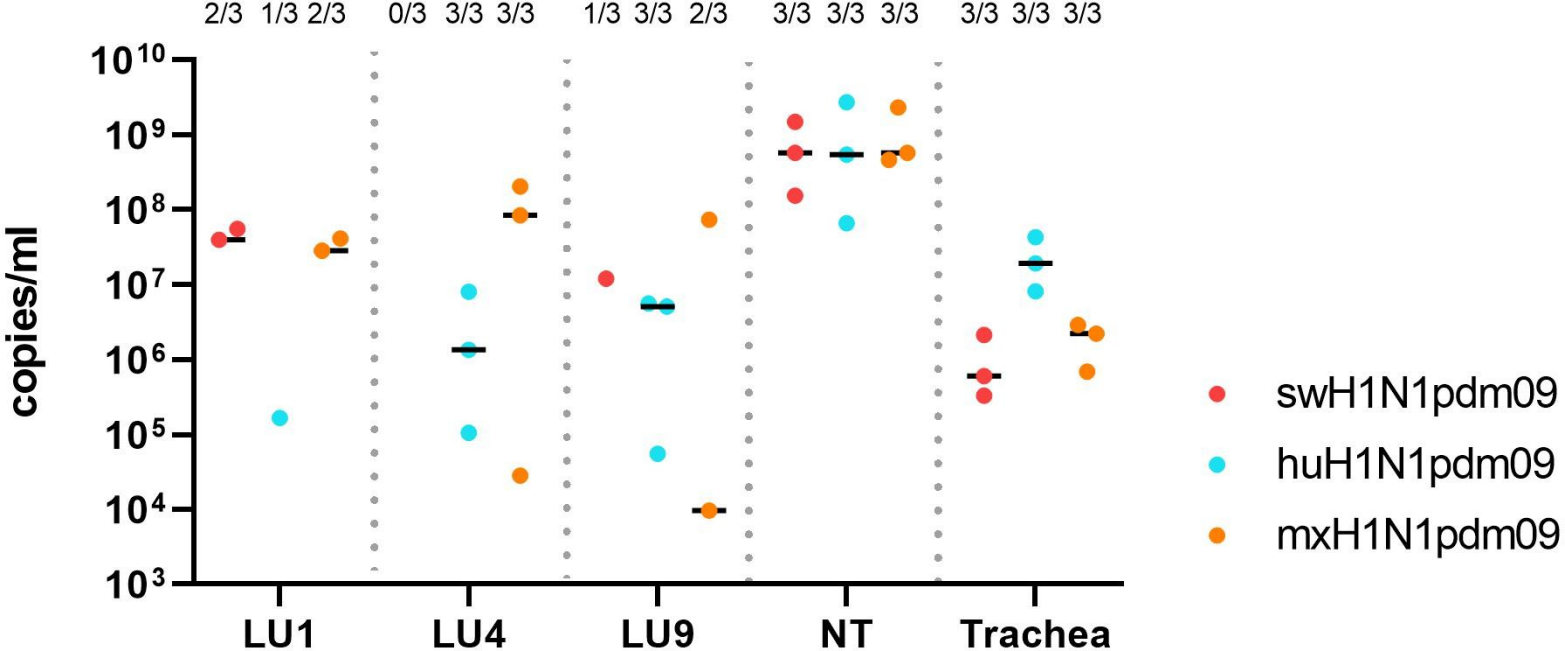
Viral load in inoculated ferret lung tissues (LU1, LU4, LU9), nasal turbinates (NT), and trachea collected at 3 DPI. The number of positive samples out of the total number of ferrets in the group (n  =  3) is presented above each cluster of dots as a fraction.

The viral load in tissues at 3 DPI, measured by RT-qPCR, from inoculated ferrets is shown in Fig 9, and summarized in S4 Table. In inoculated ferrets, the highest median viral load was detected in the nasal turbinates’. The huH1N1pmd09 group showed a higher viral load in trachea compared to the viral load in the lungs. The swH1N1pdm09 and mxH1N1pdm09 groups had the higher viral loads in the cranial lung lobes (LU1 and LU4) than in the trachea. One huH1N1pdm09 DC ferret (no. 13) tested positive for IAV by RT-qPCR in the nasal turbinates at 14 DPI (1.05 × 10^5^ copies/mL), whereas the remaining DC ferrets tested negative for IAV in all tissue samples. Infectious virus was isolated from all inoculated ferret in at least one tissue sample and in none of the DC ferrets at 14 DPI.

#### The huH1N1pdm09-infected groups showed the mildest histopathological changes

Some histopathological changes, which were considered to be unrelated to IAV infection, were found in all groups (S1 Text) and were not taken into account in the histopathological evaluation. Lesions were observed in one of three swH1N1pdm09 inoculated ferrets, one of three huH1N1pdm09 inoculated ferrets, and two of three mxH1N1pdm09 inoculated ferrets (S5 Table). The lesions ranged from acute, mild, suppurative bronchiolitis with a focal or multifocal distribution (S7A Fig) to acute, mild, necrotizing bronchointerstitial pneumonia (S7B Fig). The lesions affected <10% of the bronchioles.

Only mild pulmonary lesions were found in DC ferrets at 14 DPI. These represented mild, suppurative bronchiolitis with a focal or multifocal distribution, affecting <10% of the bronchioles (S5 Table).

In the nasal turbinates, acute, moderate, suppurative, necrotizing rhinitis was observed in all inoculated groups at 3 DPI (S7C Fig). Additionally, all inoculated ferrets presented acute, mild tracheitis at 3 DPI, with mild infiltration of neutrophils in the lamina propria and some neutrophils migrating through the tracheal epithelial cells. No lesions were observed in the nasal or tracheal tissues from huH1N1pdm09-infected DC ferrets at 14 DPI, whereas there was mild infiltration of neutrophils in the lamina propria of both tissues from mxH1N1pdm09-infected and swH1N1pdm09-infected DC ferrets.

## Discussion

The overall aim of this study was to compare the infection dynamics and pathogenesis of three strains of H1N1pdm09 in pigs and ferrets. The higher virus replication of the swH1N1pdm09 and mxH1N1pdm09 viruses in pigs indicates that these viruses are more adapted to pigs than the huH1N1pdm09 virus. Additionally, lung lesions were significantly more severe in the mxH1N1pdm09-infected group than in the huH1N1pdm09-infected group, and the clinical impact of the mxH1N1pdm09 strain in pigs was greater than that of the swH1N1pdm09 strain. These findings indicate that after at least 10 years of circulation in pigs, the swH1N1pdm virus has increased its ability to replicate in pigs but appears to have reduced pathogenicity, perhaps reflecting pathogen–host adaptation. These results support previous findings of host-adaptation of IAVs that have circulated in their hosts for at least 5 years, including human H1N1pdm09 viruses (investigated in pigs [37], and in human cells, and mice [38]), an equine H3N8 influenza [39], and an avian H5N1 influenza [40]). In contrast to another study [37], the viral lung load was highest in pigs infected with the swine-origin (sw and mx) viruses and the clinical impact of the swine-adapted H1N1pdm09 in pigs was less than that of the human-adapted H1N1pdm09 virus. Overall, however, the viral dynamics, clinical signs, and lesions corresponded to those reported by others in pigs experimentally infected with different H1N1pdm09 strains [26,41,42].

Some of the pigs showed signs of emphysema that were probably due to excessive gasping at euthanasia, a phenomenon described previously [43]. All pigs in this study tested negative for PCV2, PRRSV types 1 and 2, and *Mycoplasma hyopneumoniae*. However, because they were from a commercial sow herd, they may have harbored opportunistic bacterial pathogens that could have contributed to some of the clinical and pathological changes seen. For example, four pigs developed polyserositis (pleuritis and pericarditis) and three of these became lethargic during the study. This lethargy was probably not a response to the IAV infection but rather the result of a bacterial co-infection [44,45]. The left and right lung lobes showed different viral loads and lesions. This might be explained by the fact that the pigs were put on either the right or left side until they woke up after sedation during the inoculation.

In contrast to the findings in pigs, the huH1N1pdm09 strain replicated most efficiently and induced the lowest weight loss in inoculated and DC ferrets, whereas infection with the swH1N1pdm09 strain resulted in the lowest viral load and an intermediary weight loss. This finding supports the relevance of the ferret model in human-adapted IAV transmission studies [46,47]. The histopathological changes in inoculated and DC ferrets corresponded to those reported by others in ferrets experimentally infected with the PR8 H1N1 IAV strain [48]. Furthermore, the viral dynamics of inoculated and DC ferrets corresponded to those reported in ferrets experimentally infected with an H1N1pdm09 strain, with viral shedding being highest at the beginning of the infection and with the viral load being highest in the nasal turbinates or lungs [49,50]. In contrast to the findings of Munster et al. [50] and Belser et al. [51], who experimentally infected ferrets with A/California/07/2009 (H1N1)pdm09, we observed only a limited clinical impact in our ferret study. This could be because the strains we used have acquired host adaptations important for decreasing the pro-inflammatory responses [38,39].

In the ferret study, the highest clinical impact, the highest viral load in the lungs, the most severe lung lesions, and an intermediary total viral load were found in the mxH1N1pdm09-infected inoculated and DC ferrets, which is comparable to the results of the pig experiment. Additionally, in both animal models, the two swine-origin viruses produced the highest viral load in the lungs and more severe lung lesions when compared to the huH1N1pdm09 strain. This finding is consistent with that of Pulit-Penalosa et al. [52], who found that swine-origin H1N1 and H1N2 viruses generally produced higher viral loads in the lower respiratory tract of ferrets when compared with human-origin H1N1pdm09 strains. The higher viral load in the lungs and the higher clinical impact in ferrets inoculated with the swine-origin viruses may be explained by the fact that subsequent back-titrations of the inoculum showed that, unintentionally, ferrets in these groups received 1–1.5 log10 more virus than did those in the huH1N1pdm09 group. One study investigated the clinical signs and virus kinetics in ferrets inoculated with a low dose (10^2^ TCID50), a medium dose (10^4^ TCID50), and a high dose (10^6^ TCID50) of A/California/04/2009. The researchers found that the viral shedding, viral clearance, and greatest weight loss were delayed in the lower-dose group and delayed to an intermediate extent in the medium-dose group, as compared with the high-dose group. Furthermore, the low-dose group had the highest total viral load (AUC) as a result of increased peak viral shedding and delayed viral clearance [53]. Therefore, if the huH1N1pdm09 inoculated ferrets had received the same dose of virus as the swH1N1pdm09 and mxH1N1pdm09 inoculated groups, the viral shedding at 2 DPI, and the viral load in the tissues at 3 DPI may have been higher and they might have had a greater weight loss from 0 to 2 DPI. On the other hand, the responses of the inoculated ferrets were comparable to those found in the direct-contact ferrets. Nevertheless, the differences in the clinical impact and viral kinetics of the inoculated ferrets should be interpreted with caution due to the different inoculum doses.

The mxH1N1pdm09 virus was isolated from a pig in Mexico in 2012, but phylogenetically it is situated at a node before the first detected human virus in 2009. Hypothesizing that this strain represents an early precursor of the strain that jumped to humans, it is interesting that this virus appears to be less adapted than the swH1N1pdm09 virus to pigs but better adapted to ferrets. This in turn may indicate that the precursor H1N1pdm09 virus circulated in pigs for a limited time before jumping to humans.

In the AT ferrets, the huH1N1pdm09 virus induced the highest level of viral shedding, but, in contrast to the outcome in the inoculated and DC ferrets, infection also resulted in weight loss. Similar to the dynamic of the huH1N1pdm09 virus in pigs, shedding of the swH1N1pdm09 virus by ferrets was delayed and at a lower level than with the mxH1N1pdm09 virus. Consistent with our findings, another study found that IAV strains differ in their capacity for aerosol transmission between ferrets [54]. Infection with the swH1N1pdm09 strain resulted in positive nasal washes at 1 DPI from two of three AT ferrets. Therefore, we speculate that these viruses have the potential to adapt and be transmitted between humans, because the ferret respiratory droplet transmission model resembles transmission in humans [55], however, caution must be applied, as the findings might not demonstrate the real risk of human-to-human transmission due to the small sample size and because no infectious virus was isolated from the swH1N1pdm09 AT ferrets. Nevertheless, two sporadic cases of swine-to-human transmission of swine-adapted H1N1pdm09 strains were documented in Denmark in 2021, further emphasizing that the clade 1A.3.3.2 viruses have indeed retained their ability to infect humans despite being long adapted to swine [56,57].

This study highlights the relevance of host adaptation; however, more studies are required to determine which mutations are important for host adaptation. Additionally, further work is needed to investigate the innate immune responses to elucidate whether the different clinical outcomes are due to different regulation of the immune system by the viruses.

## Supporting information

S1 Text Supporting information. (DOCX)

S1 Fig Phylogenetic relationship between inoculum strains. Maximum likelihood tree of nucleotide H1pdm09 segments of the inoculum strains (colored red, blue, and orange), a reference strain for the 2009 pandemic (A/California/07/2009, colored green), and reference strains whose origins are described in the Methods section. Node labels represent bootstrap values.
(TIF)

S2 Fig Total viral load of pigs on days 1, 2, 3 post inoculation (DPI) visualized as the median area under the curve (AUC) and dynamics of viral shedding in the pigs. A) Comparison of the total viral loads on days 1, 2, and 3 DPI visualized as the median area under the curve (AUC), for the swH1N1pdm09, huH1N1pdm09, and mxH1N1pdm09 groups. B) Scatterplot of viral shedding detected in nasal swabs collected on DPI 1, 2, 3, 4, 7, 10, and 14. Black lines represent the median viral shedding for virus-positive samples. The number of virus-positive samples out of the total number of pigs in the group (n  =  4) is shown above each cluster of dots. If no number is indicated, all pigs in that group were virus positive.
(TIF)

S3 Fig Macroscopic appearance of swH1N1pdm09, huH1N1pdm09 and mxH1N1pdm09. Macroscopic appearance of representative lungs collected from pigs 3 days after they were inoculated with different strains of IAV. Areas with atelectasis are marked with arrows. Pigs inoculated with mxH1N1pdm09 had more atelectasis than did pigs inoculated with huH1N1pdm09 or swH1N1pdm09.
(TIF)

S4 Fig Histopathological findings in lungs of control pigs at 3 days post inoculation (DPI), in nasal mucosa and tracheal tissues at 3 DPI of infected pigs, and in tracheal tissues at 14 DPI of infected pigs. A) Lung tissue from a control pig at 3 DPI, showing organized bronchus-associated lymphoid tissue (BALT) (arrows) throughout. B) Lung tissue from a different control pig at 3 DPI, showing infiltration of macrophages (arrows) and neutrophils (arrowheads). C) Nasal mucosa from an swH1N1pdm09-infected pig at 3 DPI, showing exudation of neutrophils (arrowhead), necrosis (star), and desquamation of epithelial cells (arrow). D) Trachea from an swH1N1pdm09-infected pig at 3 DPI, showing exocytosis of neutrophils (arrowhead) and loss of cilia (arrow). E) Trachea from an mxH1N1pdm09-infected pig at 3 DPI, showing neutrophils present in the lamina propria and lamina epithelialis (arrowheads). F) Lung tissue from an huH1N1pdm09-infected pig at 14 DPI, showing macrophages (arrows) and a few neutrophils (arrowhead). H&E stained.
(TIF)

S5 Fig Body temperatures in inoculated, direct-contact (DC) and aerosol transmission (AT) ferrets. A) The change in body temperature (in°C) from baseline (0 DPI) in inoculated ferrets (n  =  3) at 2 DPI. The mean baseline temperatures in inoculated ferrets were 38.1°C, 38.4°C, and 38.4°C for the swH1N1pdm09, huH1N1pdm09, and mxH1N1pdm09 groups, respectively. B) The change in body temperature (in°C) from baseline (0 DPI) in DC ferrets (n  =  3) at 2, 5, 7, and 9 DPI. The average baseline temperatures in DC ferrets were 38.6°C, 38.7°C, and 38.5°C for the swH1N1pdm09, huH1N1pdm09, and mxH1N1pdm09 groups, respectively. C) The change in body temperatures (in°C) from baseline (0 DPI) in AT ferrets (n  =  9) at 2, 5, 7, and 9 DPI. The average baseline temperatures in AT ferrets were 38.3°C, 38.3°C, and 38.7°C for the swH1N1pdm09, huH1N1pdm09, and mxH1N1pdm09 groups, respectively. Ferrets that did not test positive for IAV at any time point during the study are marked with crosses. DPI  =  days post inoculation.
(TIF)

S6 Fig Body weight loss in inoculated, direct-contact (DC) and aerosol transmission (AT) ferrets and the total viral load of DC and AT ferrets on days post inoculation (DPI) 0, 2, 5, 7, and 9 visualized as the median area under the curve (AUC). A) Percentage body weight loss in inoculated ferrets (n  =  3) from 0 to 2 DPI. Black lines represent the median weight loss per group. B) Percentage body weight loss in DC ferrets (n  =  3) from 0 to 2, 0 to 5, 0 to 7, and 0 to 9 DPI. Black lines represent the median weight loss per group. C) Percentage body weight loss in AT ferrets (n  =  3) from 0 to 2, 0 to 5, 0 to 7, and 0 to 9 DPI. Black lines represent the median weight loss per group. Crosses in the scatterplot indicate ferrets that tested negative for IAV in nasal washes at any time point. D) Comparison of the AUCs for the swH1N1pdm09, huH1N1pdm09, and mxH1N1pdm09 DC ferret groups. E) Comparison of the AUCs for the swH1N1pdm09, huH1N1pdm09, and mxH1N1pdm09 AT ferret groups.
(TIF)

S7 Fig Histopathological changes in the lungs and nasal turbinates of inoculated ferrets. A) Lung tissue from an mxH1N1pdm09-inoculated ferret at 2 DPI, showing scant exudation of neutrophils (arrowheads). B) Lung tissue from an swH1N1pdm09- inoculated ferret at 2 DPI, showing multifocal, suppurative, necrotizing bronchiolitis (arrow) and exudation to adjacent alveoli (arrowheads). Peribronchiolar and interstitial infiltration dominated by mononuclear cells (stars) is also present. C) Nasal turbinates from an mxH1N1pdm09-inoculated ferret at 2 DPI, showing infiltration of neutrophils (arrowheads), mononuclear cells in the lamina propria, and necrosis of the nasal epithelium. Notice that only the basal cells remain. H&E stained.
(TIF)

S1 Table Specimens collected for virological and histopathological analysis. (DOCX)

S2 Table Histopathology scoring scheme. (DOCX)

S3 Table Morphological diagnoses of the lung, nasal mucosa, and tracheal lesions observed at 3 and 14 days post inoculation (DPI) and the number of affected pigs in each group. HP = histopathological score. (DOCX)

S4 Table Summary of results from inoculated ferrets. (DOCX)

S5 Table Morphological diagnoses of lung lesions in inoculated ferrets at 3 days post inoculation (DPI) and in direct contact (DC) infected ferrets at 14 DPI. The number of affected ferrets in each group is shown. (DOCX)

S1 Acknowledgements Acknowledgement table containing accession numbers of the inoculum strains and reference sequences used in S1 Fig. (XLS)
