# Supplementary material for: Experimental infection of pigs and ferrets with “pre-pandemic,” human-adapted, and swine-adapted variants of the H1N1pdm09 influenza A virus reveals significant differences in viral dynamics and pathological manifestations
Source: PLoS Pathog. 2023 Dec 4;19(12):e1011838. doi: 10.1371/journal.ppat.1011838 (PMC10721187; doi:10.1371/journal.ppat.1011838)
Supplement: S1 Text — (DOCX) [file ppat.1011838.s001.docx]

**Text S1. Supporting text.**

**Methods of the pig experiment.** Before inoculation, all pigs were sedated by intramuscular injection (0.1 mL/kg) of a Zoletil mixture consisting of one Zoletil 50 Vet (without solvent) mixed with 6.25 Rompun (20 mg/mL), 1.25 mL Ketaminol (100 mg/mL), and 2.50 mL Torbugesic (butorphanol tartrate) (10 mg/mL).

Blood was drawn from the jugular vein with a vacutainer and collected in 6-mL serum tubes (VWR International, Radnor, PA). Nasal swabs were performed by inserting a sterile cotton swab into the left nostril, rotating it through 360°, then immersing it in a tube containing 1 mL of Sigma Virocult medium (Medical Wire, Corsham, UK). Swab samples were kept at room temperature for 30 min then vortex mixed for 10 s before being transferred to 1.5-mL Eppendorf tubes and stored at −80°C until analyzed. Serum tubes were centrifuged at 1550 × *g* for 10 min at 4°C, then the serum was transferred to 1.5-mL Eppendorf tubes and stored at −20°C until analyzed. Monitoring for clinical signs such as coughing, anorexia, dyspnea, and lethargy was performed twice a day during the experiment, and NSAIDs were given to lethargic pigs. All pigs were treated with ZooLac Propaste (Chemvet A/S, Silkeborg, Denmark) to prevent diarrhea.

**Clinical signs in the pigs.** During the study, no pigs in the swH1N1pdm09 group developed a fever (temperature ≥40°C). Three pigs in the huH1N1pdm09 (nos. 9, 25, and 28) developed a fever at 1 DPI, two pigs in the mxH1N1pdm09 group (nos. 37 and 38) developed a fever at 1 DPI, one pig in that group (no. 42) had developed a fever at 2 DPI, and two pigs in that group developed a fever at 4 DPI (nos. 40, and 41). Pigs 42 and 40 also had developed fever at 4 DPI and 7 DPI, respectively. The mean body weights at 0 DPI were 10.2 kg, 9.5 kg, 9.8 kg and 9.7 kg for the control, swH1N1pdm09, huH1N1pdm09, and mxH1N1pdm09 groups, respectively.

Sneezing were observed in pigs 13 and 14 (in the swH1N1pdm09 group) at 3 DPI. Lethargy was observed at various times in three pigs: no. 18 in the swH1N1pdm09 group (at 11 DPI), no. 27 in the huH1N1pdm09 group (at 14 DPI), and no. 41 in the mxH1N1pdm09 group (at 9 DPI, 11 DPI and 13 DPI). Two of these pigs (nos. 18 and 27) were treated with meloxicam (5 mg/mL) for 1 day at 11 DPI and 12 DPI, respectively, whereas one pig (no. 41) received treatment for 4 days (from 10 to 13 DPI). Five pigs developed diarrhea for 1 or 2 days at 1–3 DPI: these five pigs comprised two from the control group (nos. 1 and 4), two from the swH1N1pdm09 group (16 and no. 17), and one from the mxH1N1pdm09 group (no. 38).

**Clinical signs in the ferrets**. One ferret from the swH1N1pdm09 group (no. 20) and one from the mxH1N1pdm09 group (no. 26) tested negative for IAV in nasal washes collected at 2, 5, 7, and 9 DPI. These animals are marked with crosses in S5C Fig and S6C Fig.

**Macroscopic and microscopic evaluation of the pigs.** Histopathological lesions characteristic of hyperleukocytosis, interlobular edema, and emphysema were present in all pigs, including the controls. Desquamated epithelial cells and macrophages in the lumens of bronchioles, focal infiltrations with mononuclear cells in the interstitium, and massive hyperplasia of bronchus-associated lymphoid tissue (BALT) were additional findings in some control pigs. Some pigs, including controls, had erythrocytes and leukocytes in the lumens of their bronchioles, probably due to aspiration of blood at euthanasia. Some of the lesions present in the controls were also found in pigs inoculated with IAV; however, these lesions were not taken into account in the histopathological grading. Furthermore, three control pigs had acute, mild, multifocal, suppurative bronchiolitis affecting <10% of the bronchioles (Table S3, S4A & S4B Figs); therefore, these lesions were not considered to be caused by IAV.

**Microscopic evaluation of the ferrets.** The lung tissue sections of some ferrets contained one or more foreign bodies with granuloma formation. The lung tissue from one ferret (no. 14) had no visible foreign bodies but had an area of massive perivasculitis and a pyogranulomatous inflammation in which giant cells and macrophages predominated, with evidence of epithelioid cells and neutrophil granulocytes too. Perivasculitis and perivascular edema were found in all ferrets, even though no other lesions were present; therefore, these lesions were not taken into account in the histopathological examination, and neither were the pulmonary edema and mineralization found sporadically in some ferrets.
