# Supplementary figures and images for: Experimental infection of pigs and ferrets with “pre-pandemic,” human-adapted, and swine-adapted variants of the H1N1pdm09 influenza A virus reveals significant differences in viral dynamics and pathological manifestations
Source: PLoS Pathog. 2023 Dec 4;19(12):e1011838. doi: 10.1371/journal.ppat.1011838 (PMC10721187; doi:10.1371/journal.ppat.1011838)

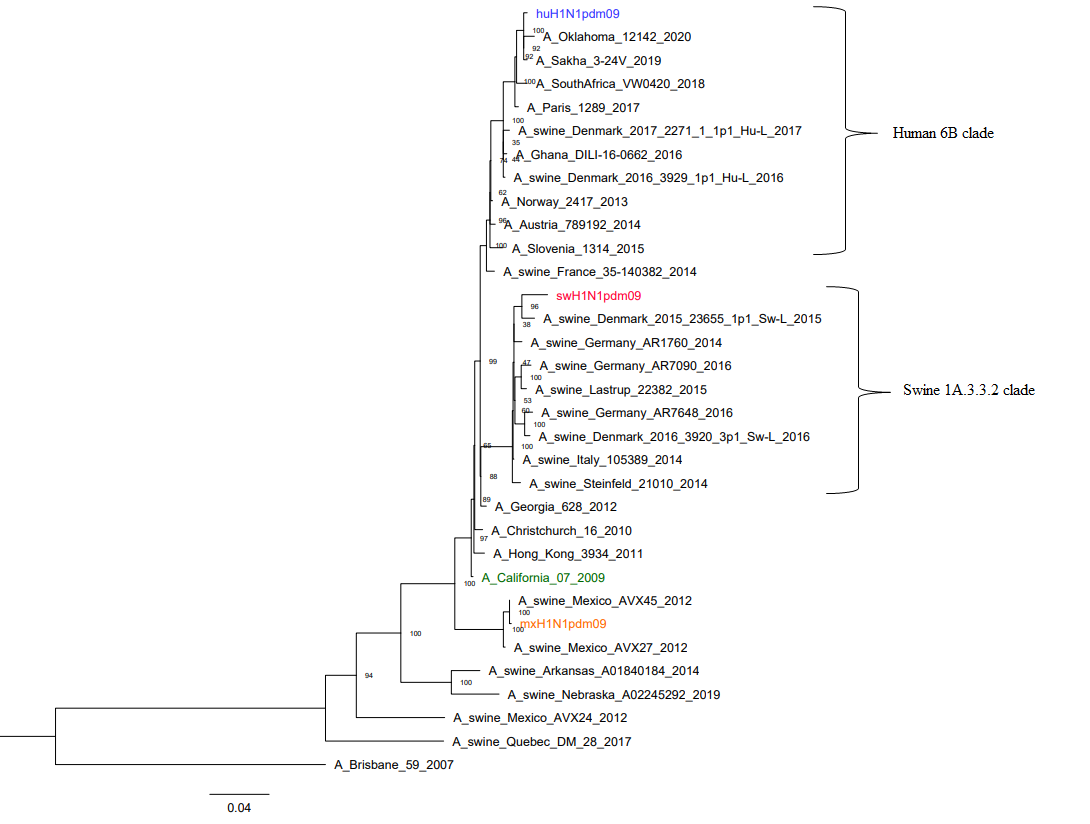

Supplement: S1 Fig — Maximum likelihood tree of nucleotide H1pdm09 segments of the inoculum strains (colored red, blue, and orange), a reference strain for the 2009 pandemic (A/California/07/2009, colored green), and reference strains whose origins are described in the Methods section. Node labels represent bootstrap values. (TIF) [file ppat.1011838.s002.tif]

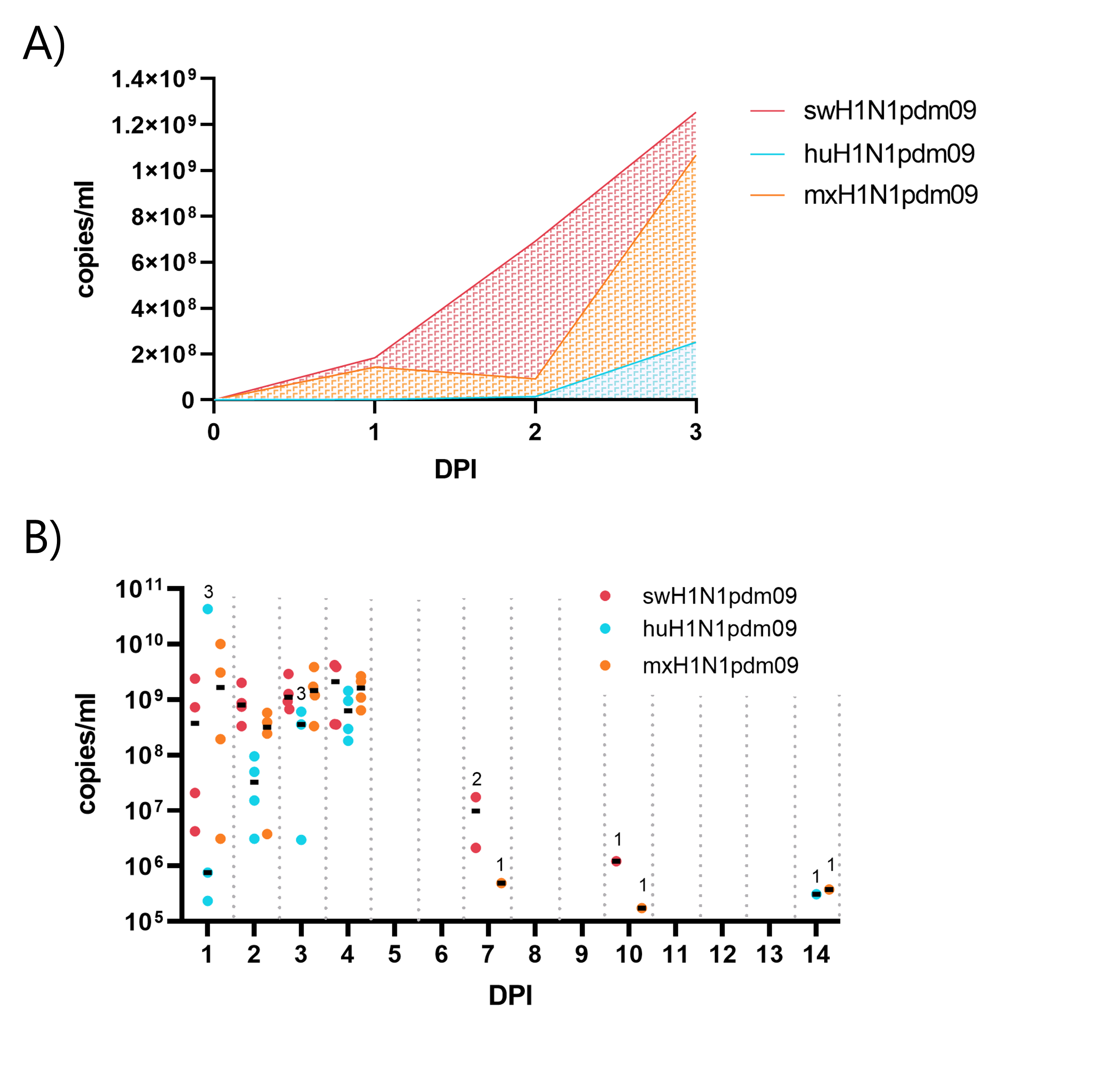

Supplement: S2 Fig — A) Comparison of the total viral loads on days 1, 2, and 3 DPI visualized as the median area under the curve (AUC), for the swH1N1pdm09, huH1N1pdm09, and mxH1N1pdm09 groups. B) Scatterplot of viral shedding detected in nasal swabs collected on DPI 1, 2, 3, 4, 7, 10, and 14. Black lines represent the median viral shedding for virus-positive samples. The number of virus-positive samples out of the total number of pigs in the group (n = 4) is shown above each cluster of dots. If no number is indicated, all pigs in that group were virus positive. (TIF) [file ppat.1011838.s003.tif]

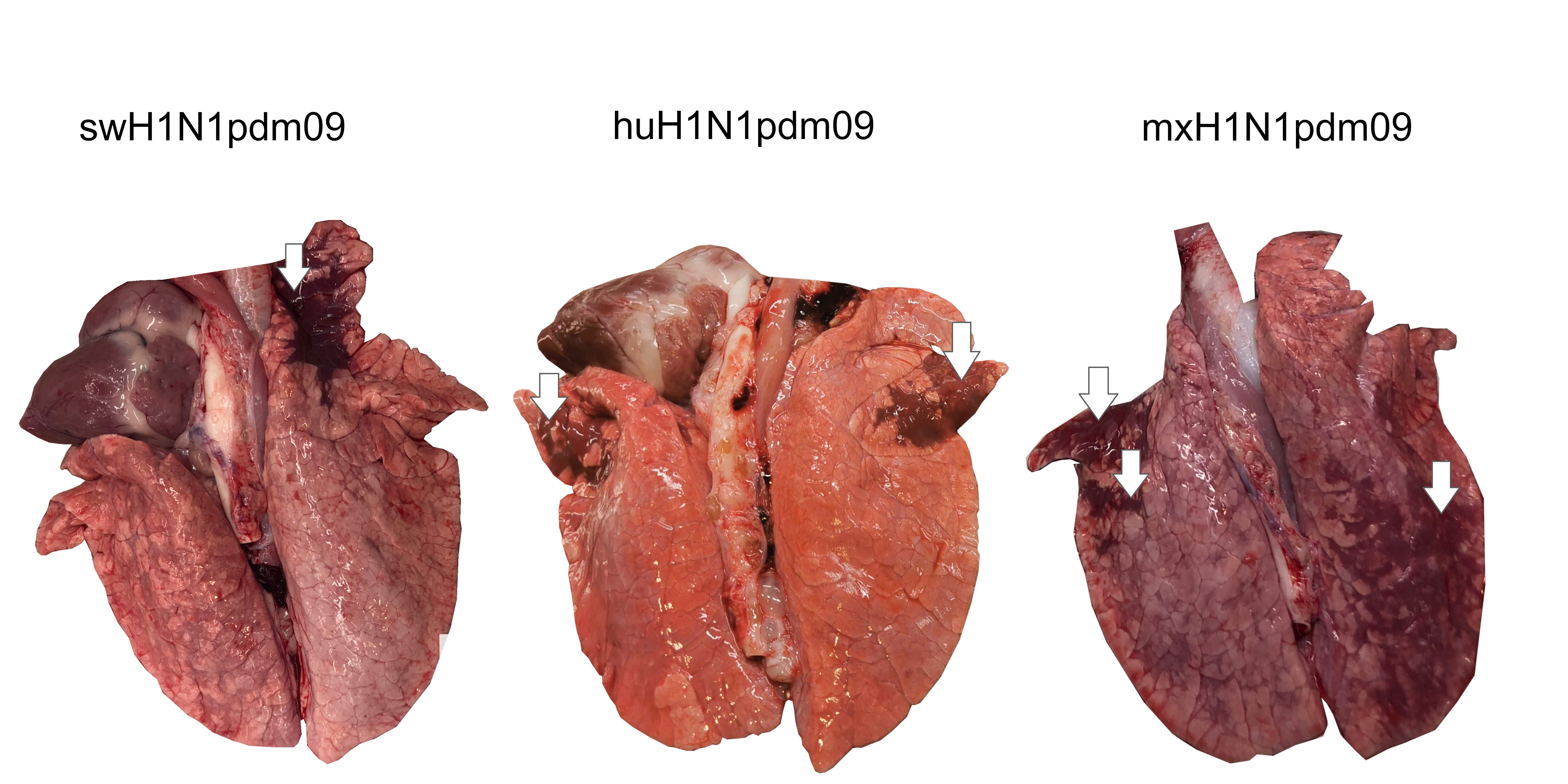

Supplement: S3 Fig — Macroscopic appearance of representative lungs collected from pigs 3 days after they were inoculated with different strains of IAV. Areas with atelectasis are marked with arrows. Pigs inoculated with mxH1N1pdm09 had more atelectasis than did pigs inoculated with huH1N1pdm09 or swH1N1pdm09. (TIF) [file ppat.1011838.s004.tif]

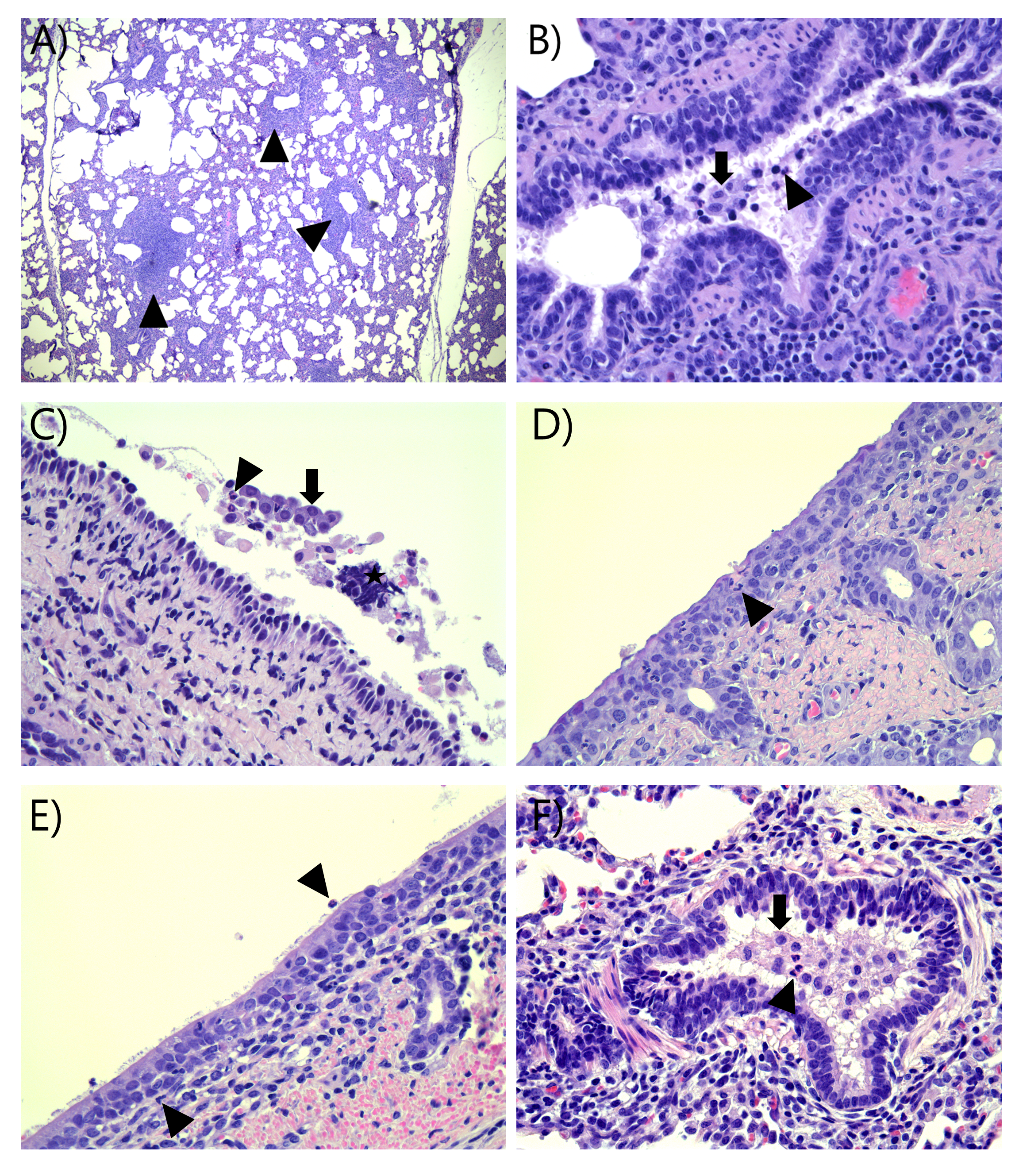

Supplement: S4 Fig — A) Lung tissue from a control pig at 3 DPI, showing organized bronchus-associated lymphoid tissue (BALT) (arrows) throughout. B) Lung tissue from a different control pig at 3 DPI, showing infiltration of macrophages (arrows) and neutrophils (arrowheads). C) Nasal mucosa from an swH1N1pdm09-infected pig at 3 DPI, showing exudation of neutrophils (arrowhead), necrosis (star), and desquamation of epithelial cells (arrow). D) Trachea from an swH1N1pdm09-infected pig at 3 DPI, showing exocytosis of neutrophils (arrowhead) and loss of cilia (arrow). E) Trachea from an mxH1N1pdm09-infected pig at 3 DPI, showing neutrophils present in the lamina propria and lamina epithelialis (arrowheads). F) Lung tissue from an huH1N1pdm09-infected pig at 14 DPI, showing macrophages (arrows) and a few neutrophils (arrowhead). H&E stained. (TIF) [file ppat.1011838.s005.tif]

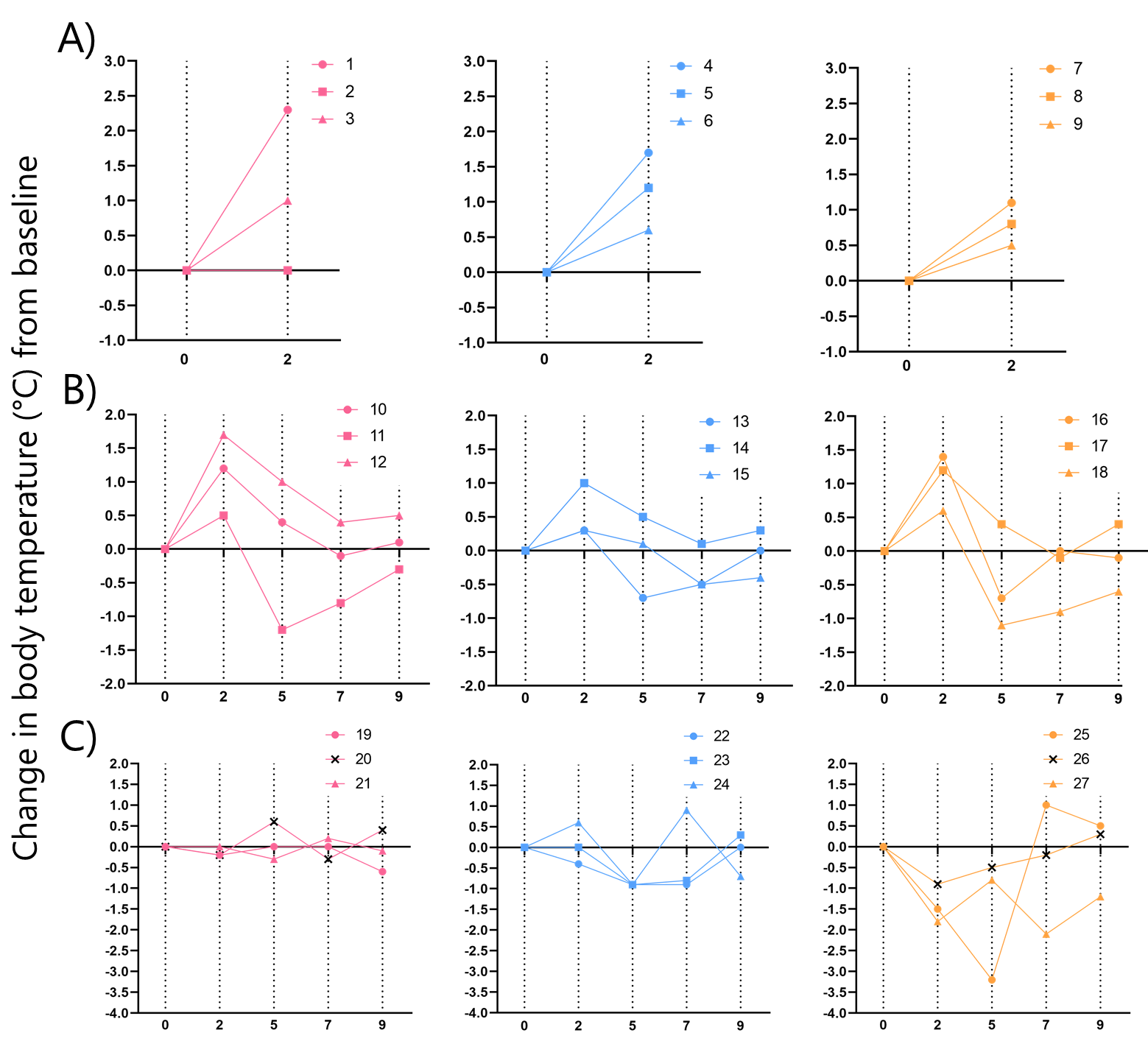

Supplement: S5 Fig — A) The change in body temperature (in°C) from baseline (0 DPI) in inoculated ferrets (n = 3) at 2 DPI. The mean baseline temperatures in inoculated ferrets were 38.1°C, 38.4°C, and 38.4°C for the swH1N1pdm09, huH1N1pdm09, and mxH1N1pdm09 groups, respectively. B) The change in body temperature (in°C) from baseline (0 DPI) in DC ferrets (n = 3) at 2, 5, 7, and 9 DPI. The average baseline temperatures in DC ferrets were 38.6°C, 38.7°C, and 38.5°C for the swH1N1pdm09, huH1N1pdm09, and mxH1N1pdm09 groups, respectively. C) The change in body temperatures (in°C) from baseline (0 DPI) in AT ferrets (n = 9) at 2, 5, 7, and 9 DPI. The average baseline temperatures in AT ferrets were 38.3°C, 38.3°C, and 38.7°C for the swH1N1pdm09, huH1N1pdm09, and mxH1N1pdm09 groups, respectively. Ferrets that did not test positive for IAV at any time point during the study are marked with crosses. DPI = days post inoculation. (TIF) [file ppat.1011838.s006.tif]

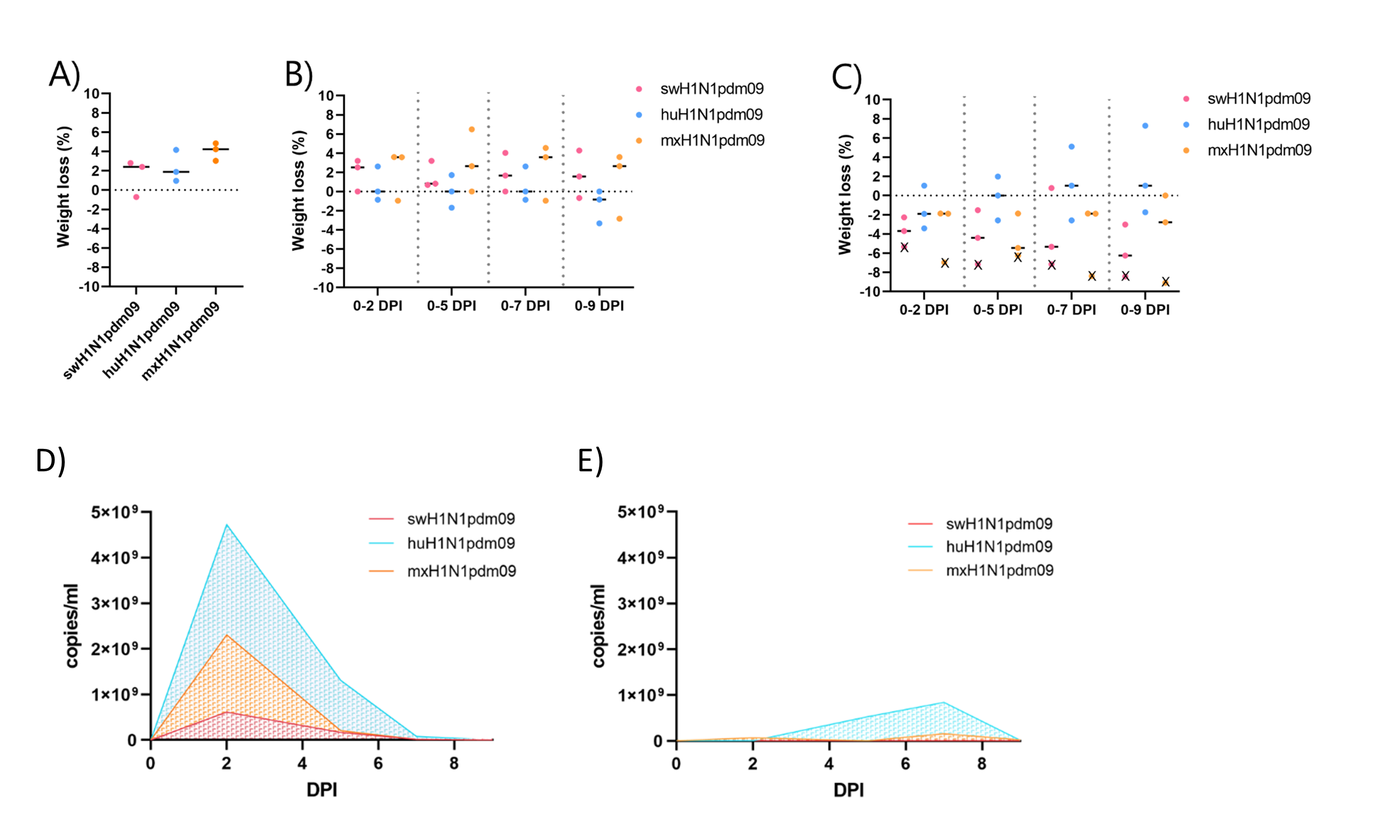

Supplement: S6 Fig — A) Percentage body weight loss in inoculated ferrets (n = 3) from 0 to 2 DPI. Black lines represent the median weight loss per group. B) Percentage body weight loss in DC ferrets (n = 3) from 0 to 2, 0 to 5, 0 to 7, and 0 to 9 DPI. Black lines represent the median weight loss per group. C) Percentage body weight loss in AT ferrets (n = 3) from 0 to 2, 0 to 5, 0 to 7, and 0 to 9 DPI. Black lines represent the median weight loss per group. Crosses in the scatterplot indicate ferrets that tested negative for IAV in nasal washes at any time point. D) Comparison of the AUCs for the swH1N1pdm09, huH1N1pdm09, and mxH1N1pdm09 DC ferret groups. E) Comparison of the AUCs for the swH1N1pdm09, huH1N1pdm09, and mxH1N1pdm09 AT ferret groups. (TIF) [file ppat.1011838.s007.tif]

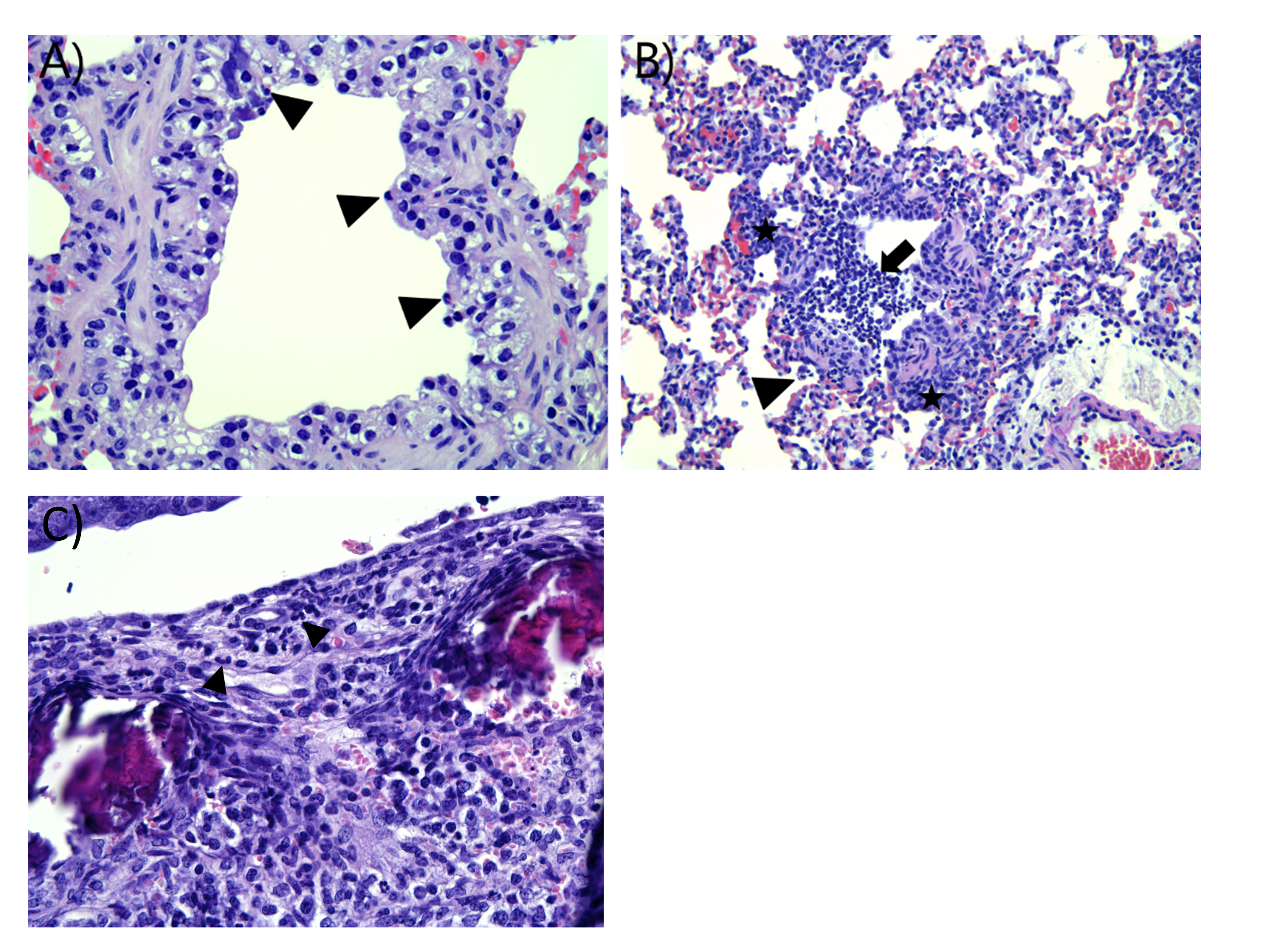

Supplement: S7 Fig — A) Lung tissue from an mxH1N1pdm09-inoculated ferret at 2 DPI, showing scant exudation of neutrophils (arrowheads). B) Lung tissue from an swH1N1pdm09- inoculated ferret at 2 DPI, showing multifocal, suppurative, necrotizing bronchiolitis (arrow) and exudation to adjacent alveoli (arrowheads). Peribronchiolar and interstitial infiltration dominated by mononuclear cells (stars) is also present. C) Nasal turbinates from an mxH1N1pdm09-inoculated ferret at 2 DPI, showing infiltration of neutrophils (arrowheads), mononuclear cells in the lamina propria, and necrosis of the nasal epithelium. Notice that only the basal cells remain. H&E stained. (TIF) [file ppat.1011838.s008.tif]
