# Supplementary material for: Experimental infection of pigs and ferrets with “pre-pandemic,” human-adapted, and swine-adapted variants of the H1N1pdm09 influenza A virus reveals significant differences in viral dynamics and pathological manifestations
Source: PLoS Pathog. 2023 Dec 4;19(12):e1011838. doi: 10.1371/journal.ppat.1011838 (PMC10721187; doi:10.1371/journal.ppat.1011838)
Supplement: S1 Table — (DOCX) [file ppat.1011838.s009.docx]

| S1 Table. Specimens collected for virological and histopathological analysis | | |
| --- | --- | --- |
| Organ | **Pigs** | **Ferrets (inoculated and DC*)** |
| Heart | X |  |
| Liver | X |  |
| Spleen | X |  |
| Nasal mucosa/nasal turbinates | X | X |
| Nasopharynx | X |  |
| Tonsilla palatina | X | X |
| Mediastinal lymph node | X |  |
| Tracheobronchial lymph node | X |  |
| Upper part of trachea (UT) | X | X (middle part) |
| Lower part of trachea (LT) | X |  |
| Lobus sinister cranialis pars cranialis (LU1) | X |  |
| Lobus sinister cranialis (LU1) |  | X |
| Lobus dexter cranialis (LU4) | X | X |
| Lobus dexter caudalis (LU9) | X | X |
| *DC = direct-contact ferret. |  |  |
