# Supplementary material for: Experimental infection of pigs and ferrets with “pre-pandemic,” human-adapted, and swine-adapted variants of the H1N1pdm09 influenza A virus reveals significant differences in viral dynamics and pathological manifestations
Source: PLoS Pathog. 2023 Dec 4;19(12):e1011838. doi: 10.1371/journal.ppat.1011838 (PMC10721187; doi:10.1371/journal.ppat.1011838)
Supplement: S2 Table — (DOCX) [file ppat.1011838.s010.docx]

**S2 Table. Histopathology scoring scheme**

The following measures of inflammation were assessed: A) the percentage of bronchial/bronchiolar sites showing exudate and periluminal infiltration in the whole-lung section; B) the severity of luminal exudate in the bronchi/bronchioles; and C) the degree of infiltration in the alveoli. The maximum possible score was 7.

1. Peribronchial/peribronchiolar infiltrates (percentage of sites)^a^
   1. None
   2. Few (<10%)
   3. Many (10–50%)
   4. Majority or all (>50%)
2. Bronchiolar luminal exudate^b^
   1. None
   2. Minimal
   3. Heavy
3. Alveolar infiltrates^c^
   1. None
   2. Minimal
   3. Heavy
4. Occasional organized bronchial associated lymphatic tissue (BALT) was seen in controls.
5. Minimal = less than 25% of the lumen occluded by neutrophils; heavy = more than >25% of the lumen occluded by neutrophils.
6. Minimal = patchy alveolar infiltration; heavy = patchy and confluent alveolar infiltrates.
