# Supplementary material for: Experimental infection of pigs and ferrets with “pre-pandemic,” human-adapted, and swine-adapted variants of the H1N1pdm09 influenza A virus reveals significant differences in viral dynamics and pathological manifestations
Source: PLoS Pathog. 2023 Dec 4;19(12):e1011838. doi: 10.1371/journal.ppat.1011838 (PMC10721187; doi:10.1371/journal.ppat.1011838)
Supplement: S3 Table — (DOCX) [file ppat.1011838.s011.docx]

**S3 Table**. **Morphological diagnoses of the lung, nasal mucosa, and tracheal lesions observed at 3 and 14 days post inoculation (DPI) and the number of affected pigs in each group**. HP = histopathological score.

| Tissue | DPI | HP | Description | Control | swH1N1pdm09 | huH1N1pdm09 | mxH1N1pdm09 |  |  |
| --- | --- | --- | --- | --- | --- | --- | --- | --- | --- |
| Lungs | 3 | 0 | No lesions | 3/6 | 1/8 | 2/8 | – |  |  |
|  |  | 2 | Acute, mild bronchiolitis with scant exudation of neutrophils; affecting <10% of the bronchioles (S4B Fig) | 3/6 | 0/8 | 1/8^1^ | – |  |  |
|  |  | 3 | Acute, mild bronchiolitis with scant exudation of neutrophils; affecting 10% –50% of the bronchioles | – | 1/8 | 1/8 | – |  |  |
|  |  | 4 –6 | Subacute, mild bronchointerstitial pneumonia^a^ (6A Fig) | – | 3/8 | 3/8 | 6/8 |  |  |
|  |  | 7 | Subacute, moderate bronchointerstitial pneumonia^b^ (6B Fig) | – | 3/8^2^ | 1/8 | 2/8 |  |  |
|  | 14 | – | Lack of epithelium in some of the terminal bronchioles | – | 1/4 | 0/4 | 2/4 |  |  |
|  |  | – | Acute, mild, bronchiolitis with infiltration of macrophages and neutrophils; affecting <10% of the bronchioles (S4F Fig) | – | 2/4^1^ | 3/4^1^ | 2/4^1^ |  |  |
|  |  | – | Acute, fibrinous, suppurative pleuritis | – | 1/4 | 1/4 | 1/4 |  |  |
| Nasal mucosa | 3 | – | No lesions observed | 1/1 | 0/2 | 0/2 | 0/2 |  |  |
|  |  | – | Acute, moderate, suppurative, necrotizing rhinitis (S4C Fig) | 0/1 | 2/2 | 2/2^3^ | 2/2 |  |  |
|  | 14 | – | No lesions observed | – | 2/2 | 2/2 | 0/2 |  |  |
|  |  | – | Mild infiltration and exudation of neutrophils | – | 0/2 | 0/2 | 2/2 |  |  |
| Trachea | 3 | – | No lesions observed | 1/1 | 0/2 | 0/2 | 0/2 |  |  |
|  |  | – | Acute, mild, tracheitis (S4E Fig) | 0/1 | 0/2 | 2/2 | 1/2^4^ |  |  |
|  |  | – | Acute, moderate, necrotizing tracheitis (S4D Fig) | 0/1 | 2/2 | 0/2 | 0/2 |  |  |
|  | 14 | – | No lesions observed | – | 2/2 | 2/2 | 0/2 |  |  |
|  |  | – | Hyperplasia of lamina epithelialis | – | 0/2 | 0/2 | 2/2 |  |  |

^a^ Bronchiolitis/bronchitis with varying amounts of exudation and +/− necrosis of the bronchiolar epithelium, and varying amounts of infiltration in the alveoli; affecting 10−50% of the bronchioles.
^b^ Necrotizing bronchiolitis/bronchitis with massive exudation and patchy and confluent infiltrations in the alveoli. ^1^ These lesions were also found in control pigs and are, therefore, not considered to be related to the experimental infection.
^2^ Necrosis of the bronchiolar epithelium was not found in one severe case (pig 14).
^3^ Pig no. 28 from the huH1N1pdm09 group had lesions compatible with porcine cytomegalovirus infection, with massive infiltration around and in the nasal glands, necrotic nasal glands, and inclusion bodies.
^4^ Only pig no. 31 from the mxH1N1pdm09 group had these lesions, whereas the other pig showed hyperplasia of the lamina epithelialis.
