# Supplementary material for: Experimental infection of pigs and ferrets with “pre-pandemic,” human-adapted, and swine-adapted variants of the H1N1pdm09 influenza A virus reveals significant differences in viral dynamics and pathological manifestations
Source: PLoS Pathog. 2023 Dec 4;19(12):e1011838. doi: 10.1371/journal.ppat.1011838 (PMC10721187; doi:10.1371/journal.ppat.1011838)
Supplement: S4 Table — (DOCX) [file ppat.1011838.s012.docx]

**S4 Table**. **Summary of results from inoculated (inoculated) ferrets.**

|  | | |  |  |  |
| --- | --- | --- | --- | --- | --- |
|  | | |  | **Group** |  |
|  | | | **swH1N1pdm09** | **huH1N1pdm09** | **mxH1N1pdm09** |
| Viral shedding^a^ | Copies/ml | | 5.79 × 10^8^  (3.19 × 10^7^–9.66×10^8^) | 3.64 × 10^9^  (2.74–6.65 × 10^9^) | 1.67 × 10^9^  (1.65–2.23 × 10^9^) |
|  | Viral titer | | 6.31 × 10^5^  (3.95 × 10^5^ –6.31 × 10^5^ ) | 1.00 × 10^7^  (5.00 × 10^6^ –1.29 × 10^7^) | 6.31 × 10^5^  (4.74 × 10^5^ –8.22 × 10^6^) |
| Clinical impact^b^ | | Weight loss (%) | 2.4 (0.8 – 2.6) (2/3) | 1.9 (1.4 – 3.0) (3/3) | 4.2 (3.6 – 4.5)  (3/3) |
|  |  | Temp. change (°C) | +1.0°C  (0.5 – 1.7°C) | +1.2°C  (0.9 – 1.5°C) | +0.8°C  (0.7 – 1.0°C) |
| Viral load in tissues^c^ | | Nasal turbinates | 5.75 × 10^8^  (3.65 × 10^8^ – 1.04 × 10^9^) | 5.46 × 10^8^  (3.06 × 10^8^ – 1.65 × 10^9^) | 5.73 × 10^8^  (5.20 × 10^8^ – 1.46 × 10^9^) |
|  |  | Trachea | 6.01 × 10^5^  (4.66 × 10^5^ –1.37 × 10^6^) | 1.93 × 10^7^  (1.37 × 10^7^ – 3.12 × 10^7^) | 2.21 × 10^6^  (1.45 × 10^6^ – 2.55 × 10^6^) |
|  |  | LU1 | 4.75 × 10^7 [1]^  (4.36 –5.15 × 10^7^) | 1.66  × 10^5^ ^[3]^ | 3.49 × 10^7 [4]^  (3.17 –3-81 × 10^7^) |
|  |  | LU4 | - | 1.35 × 10^6^  (7.25 × 10^5^ – 4.72 × 10^6^) | 8.39 × 10^7^  (4.20× 10^7^ – 1.45 × 10^8^) |
|  |  | LU9 | 1.21 × 10^7^ ^[2]^ | 5.07 × 10^6^  (2.56× 10^6^ – 5.34 × 10^6^) | 9.67 × 10^3 [5]^  (4.83 × 10^3^ – 3.70 × 10^7^) |
| Transmission^d^ | | DC-T | 3/3 \| 3/3 | 3/3 \| 3/3 | 3/3 \| 3/3 |
|  |  | AT-T | 2/3 \| 0/3 | 3/3 \| 0/3 | 2/3 \| 1/3 |

^a^ Median viral shedding (25%; 75% percentile) in nasal washes collected at 2 DPI. Copies/ml was measured by reverse-transcriptase (RT)-qPCR and the viral titer was measured by using a Median Tissue Culture Infectious Dose (TCID50) assay.
^b^ Weight loss: the median percentage weight loss (25%; 75% percentile) from day 0 to day 2 post inoculation. The number of ferrets that displayed weight loss over the total number of ferrets is shown in parentheses.
Temp. change: the median increase in body temperature (25%; 75% percentile) from baseline (the temperature at 0 DPI) at 2 DPI.
^c^ Viral load in tissues: the median viral copies/mL (25%; 75% percentile) in tissues collected at 3 DPI measured by RT-qPCR. ^[1]^ One ferret (no. 3) tested negative for IAV. ^[2]^ Two ferrets (nos. 2 and 3) tested negative for IAV. ^[3]^ Two ferrets (nos. 5 and 6) tested negative for IAV. ^[4]^ One ferret (no. 8) tested negative for IAV (no. 8). ^[5]^ One ferret (no. 7) tested negative for IAV.
^d^ DC-T: the number of direct-contact ferrets that tested positive for IAV at least once during the study over the total number of ferrets. AT-T: the number of aerosol transmission ferrets that tested positive for IAV at least once during the study over the total number of ferrets. Measured by the two methods RT-qPCR | TCID50.
