# Supplementary material for: Experimental infection of pigs and ferrets with “pre-pandemic,” human-adapted, and swine-adapted variants of the H1N1pdm09 influenza A virus reveals significant differences in viral dynamics and pathological manifestations
Source: PLoS Pathog. 2023 Dec 4;19(12):e1011838. doi: 10.1371/journal.ppat.1011838 (PMC10721187; doi:10.1371/journal.ppat.1011838)
Supplement: S5 Table — (DOCX) [file ppat.1011838.s013.docx]

**S5 Table. Morphological diagnoses of lung lesions in inoculated ferrets at 3 days post inoculation (DPI) and in direct contact (DC) infected ferrets at 14 DPI.** The number of affected ferrets in each group is shown.

| **Infection route** | **Group** | **Acute, mild, focal or multifocal suppurative bronchiolitis** | **Acute, mild, necrotizing, bronchointerstitial pneumonia** |
| --- | --- | --- | --- |
| Inoculated ferrets | swH1N1pdm09 | 0/3 | 1/3 |
|  | huH1N1pdm09 | 1/3 | 0/3 |
|  | mxH1N1pdm09 | 1/3 | 1/3 |
| DC ferrets | swH1N1pdm09 | 3/3 | 0/3 |
|  | huH1N1pdm09 | 1/3 | 0/3 |
|  | mxH1N1pdm09 | 3/3 | 0/3 |
